# Phenolic Compounds and Anthocyanins in Legumes and Their Impact on Inflammation, Oxidative Stress, and Metabolism: Comprehensive Review

**DOI:** 10.3390/molecules30010174

**Published:** 2025-01-04

**Authors:** Rocio Guadalupe Hernández-Ruiz, Xochitl Citalli Olivares-Ochoa, Yahatziri Salinas-Varela, David Guajardo-Espinoza, Luis Gustavo Roldán-Flores, Edgar Alfonso Rivera-Leon, Andres López-Quintero

**Affiliations:** 1 Doctorado en Ciencias de la Nutrición Traslacional, Centro Universitario de Ciencias de la Salud (CUCS), Universidad de Guadalajara (UdeG), Guadalajara 44340, Jalisco, Mexico; rocio.hernandez9558@alumnos.udg.mx (R.G.H.-R.); xochitl.olivares2305@alumnos.udg.mx (X.C.O.-O.); patricia.salinas3709@alumnos.udg.mx (Y.S.-V.); 2 Licenciatura en Médico Cirujano y Partero, CUCS, UdeG, Guadalajara 44340, Jalisco, Mexico; 3 Instituto de Nutrigenética y Nutrigenómica Traslacional, CUCS, UdeG, Guadalajara 44340, Jalisco, Mexico; edgar.rleon@academicos.udg.mx; 4 Departamento de Ciencias de la Salud, Centro Universitario de los Altos (CUAltos), UdeG, Tepatitlán de Morelos 47620, Jalisco, Mexico

**Keywords:** phenolic compounds, anthocyanins, legumes, inflammation, metabolism

## Abstract

Inflammation, oxidative stress, and metabolic diseases are intricately linked in a complex, self-reinforcing relationship. Inflammation can induce oxidative stress, while oxidative stress can trigger inflammatory responses, creating a cycle that contributes to the development and progression of metabolic disorders; in addition, these effects can be observed at systemic and local scales. Both processes lead to cellular damage, mitochondrial dysfunction, and insulin resistance, particularly affecting adipose tissue, the liver, muscles, and the gastrointestinal tract. This results in impaired metabolic function and energy production, contributing to conditions such as type 2 diabetes, obesity, and metabolic syndrome. Legumes are a good source of phenolic compounds and anthocyanins that exert an antioxidant effect—they directly neutralize reactive oxygen species and free radicals, reducing oxidative stress. In vivo, in vitro, and clinical trial studies demonstrate that these compounds can modulate key cellular signaling pathways involved in inflammation and metabolism, improving insulin sensitivity and regulating lipid and glucose metabolism. They also exert anti-inflammatory effects by inhibiting proinflammatory enzymes and cytokines. Additionally, anthocyanins and phenolics may positively influence the gut microbiome, indirectly affecting metabolism and inflammation.

## 1. Introduction

Many metabolic diseases are associated with oxidative stress and inflammation through complex mechanisms, and metabolic dysfunction may be associated with overconsumption of nutrients, especially simple carbohydrates and fats. Chronic nutrient excess triggers oxidative stress, which is characterized by an imbalance between reactive oxygen species (ROS) production and antioxidant defense mechanisms. Oxidative stress induces inflammatory responses through activation of redox-sensitive transcription factors, such as nuclear factor-κB (NF-κB), which upregulates the secretion of proinflammatory cytokines, including tumor necrosis factor-α (TNF-α) and interleukin-6 (IL-6), to name a few [1]. These inflammatory mediators alter metabolic homeostasis by impairing insulin signaling, compromising mitochondrial function, inducing endoplasmic reticulum stress, and promoting adipose tissue dysfunction. A sustained inflammatory state promotes a self-reinforcing cycle, and oxidative stress further stimulates inflammatory responses, progressively compromising cellular metabolism and contributing to the development of metabolic syndrome, type 2 diabetes, obesity, and others [2,3,4]. In the last decade, interest in foods containing antioxidant and anti-inflammatory properties has increased. Functional foods are an innovative and current proposal, and when consumed in a daily diet, they could benefit different diseases by improving altered metabolic parameters, including glucose, lipid profile, and insulin sensitivity [5].

Fruits and seeds that belong to the Fabaceae or Leguminosae families are known as legumes [6]. There is a wide variety around the world, and the most common and accepted are beans (*Phaseolus vulgaris*), chickpeas (*Cicer arietinum* L.), soybeans (*Glycine max*), and lentils (*Lens culinaris*). Overall, they have valuable benefits in areas such as nutrition, health, food security, and climate change [7].

Legumes are high in protein and fiber, both soluble and insoluble, and they also provide bioactive compounds or phytochemicals that could have a beneficial effect [8]. Some bioactive compounds of health relevance are peptides, polysaccharides, and phenolic compounds, such as catechins, kaempferol, and flavonoids, and within this last group are the anthocyanins [9]. Anthocyanins are water-soluble plant pigments known to be responsible for distinctive purple, red, and blue coloration, being present in flowers, fruits, vegetables, and legumes that have this spectrum of colors [10,11].

The United States Department of Agriculture (USDA) reports anthocyanins content in some fruits, vegetables, and legumes, and the latter are recognized as an excellent anthocyanins source, as well as general phenolic compounds, in addition to being accessible, low-cost, and environmentally sustainable foods, which makes them an excellent option to include in the daily diet [12]. So, the current comprehensive review aims to summarize the latest information about anthocyanins and phenolic compounds in legumes, including their metabolism, bioavailability, microbiota transformation, and nutrigenomic effect, focused on antioxidant and anti-inflammatory capacity.

## 2. Materials and Methods

In the present review, we focused on recent in vitro, in vivo, and clinical trial studies assessing the effect of legume bioactive compounds and their therapeutic potential for mitigating oxidative stress, regulating inflammation, and examining possible molecular mechanisms by which they exert their function against a variety of metabolic diseases. First, a preliminary search was carried out in the PubMed database. To identify the state-of-the-art, keywords, such as “Anthocyanins”, “Oxidative stress” [Mesh], “Inflammation”, and “Legumes”, were used. Subsequently, the search was structured as follows: ((anthocyanins [Mesh]) AND (chemistry)) AND (bioavailability), filtered by revisions and a 10-year period. Finally, the searches for each legume were performed as follows: (anthocyanins [Mesh]) AND (bean), (anthocyanins [Mesh]) AND (soybean), (anthocyanins [Mesh]) AND (lentil), (anthocyanins [Mesh]) AND (chickpea), (anthocyanins [Mesh]) AND (cowpea), (anthocyanins [Mesh]) AND (alfalfa)), with a 10-year period and simultaneous “clinical trial” filter. In addition, relevant citation lists of review articles were searched. No information was found for cowpea and alfalfa entries.

## 3. Anthocyanin’s Chemical Structure

Flavonoids are a group of secondary metabolites to which anthocyanins belong. They are naturally synthesized in plants by the phenylpropanoid pathway, and their effect depends on their absorption and bioavailability [10]. Anthocyanins consist of a typical C6-C3-C6 structural backbone (flavylium cation), having a heterocyclic ring (C-ring) in the center, and two aromatics (A-ring and B-ring) on the sides, bonded by a carbon–carbon bridge. The radical scavenging activity of these molecules is associated with the number and position of hydroxyl groups, as well as other substituents on the rings of this structure [13,14]. In nature, they are found in glycosylated form. The most common monosaccharides that bind at C3, C5, or C7 positions are glucose, galactose, arabinose, and xylose [11,15,16]. They receive their names according to the position and the number of methoxy and hydroxyl groups. There are more than 600 types of anthocyanins in the plant kingdom known to humankind; although, cyanidin, peonidin, malvidin, petunidin, pelargonidin, and delphinidin have been studied the most and are found in higher concentrations [17].

**Figure 1:**
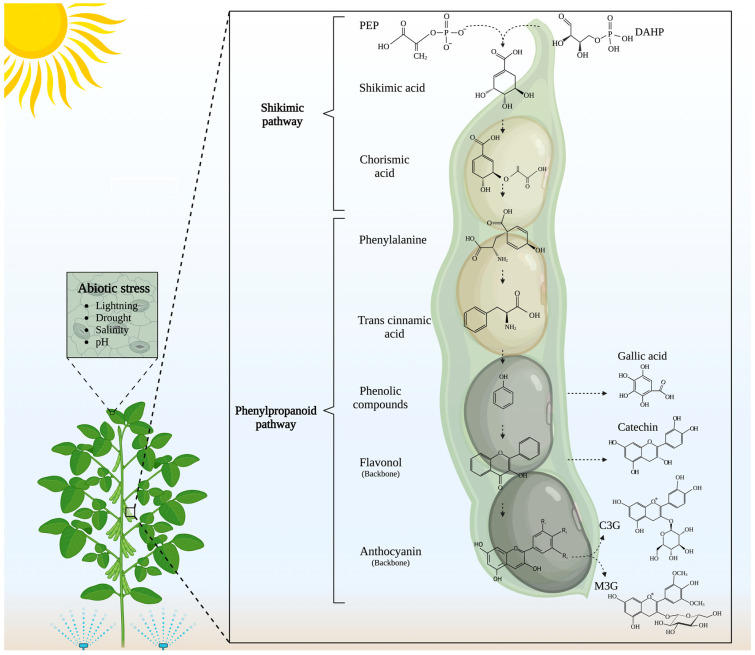
Overview of phenolic compounds and anthocyanins synthesis pathways. Shikimic acid and phenylpropanoid pathways are the main biochemical pathways involved in the synthesis of anthocyanins and phenolic compounds. Thousands of different molecules comprise the family of phenolic compounds that play important roles within the plant, encompassing pigmentation, antioxidants, and others [9]. Anthocyanins, pigments in the flavonoid subclass of phenylpropanoids, are induced in plant vegetative tissues under various abiotic stresses, such as drought, salinity, extreme temperatures, and others. In this sense, a dysregulation/modification of these conditions may lead to different concentrations of these molecules [21]. The biosynthesis of anthocyanins involves the shikimate pathway, located in the chloroplast, which comprises seven enzymatic steps, starting with the condensation of phosphoenolpyruvic acid (PEP) from glycolysis and D-erythrose-4-phosphate (DAHP) from the pentose phosphate cycle, producing as a final product chorismic acid. This acid is an important precursor for the synthesis of L-phenylalanine, essential for phenylpropanoid pathways [22]. The phenylpropanoid pathway involves three enzymatic steps: first, the deamination by phenylalanine ammonia-lyase to the trans-cinnamic acid, which then is hydrolyzed via the cinnamic acid 4-hydroxylase, resulting in 4-coumarate, which by the action of 4-coumarate-CoA ligase is converted to 4-coumaroyl-CoA. This serves as a precursor for several phenolic compounds, with gallic acid being one of the most common. The phenylalanine ammonia-lyase family is involved in the biosynthesis of flavonoids (e.g., catechin) and anthocyanins [21]. The basic carbon skeleton of anthocyanins is shown as an example; however, the most abundant anthocyanins in legumes are cyanidin-3-O-glucoside (C3G) and malvidin-3-O-glucoside (M3G) [13,15]. Created with BioRender.

Anthocyanins protect the legume plant from environmental damage due to their light absorption capacity, preventing UV-induced damage. Anthocyanins exist in a flavylium cation form under acidic conditions, where the oxygen in the heterocyclic ring (C ring) carries a positive charge. The positively charged oxygen atom confers to anthocyanins a significant antioxidant capacity, stabilizing free radicals, via hydrogen donation [18,19,20]. However, the same chemical structure that gives them the antioxidant capacity also makes them highly unstable, susceptible to changes in temperature, light, oxygen, and pH, compared to other polyphenols. For this reason, developing strategies to improve their stability is one of the most relevant challenges for researchers in the area [15] (Figure 1).

## 4. Metabolism and Bioavailability

Anthocyanins’ metabolism is similar to many phenolic compounds. After ingestion, in the oral cavity, salivary amylase partially degrades anthocyanins at a pH of 5.6–7.9. When they reach the stomach (pH 1.5–3.5), they are rapidly absorbed, as they are molecules capable of permeating the gastric mucosa. The proposed mechanism for this process is by bilitranslocase, an organic anion carrier, expressed in the gastric epithelium [16,23].

Once in the small intestine, the pH becomes neutral or semi-alkaline (pH 7.5–8.0), which decreases their stability and converts them into chalcone and hemiketal. The small intestine is considered the primary anthocyanin absorption site, and hydrolysis and oxidation-reduction reactions are carried out here. Moreover, some in vitro studies have shown that the highest absorption place is the jejunum, by hydrolytic enzymes; in the duodenum, there is a minimal absorption, and it is considered null in the ileum [24]. Only aglycones or non-glycosylated anthocyanins can be absorbed by passive diffusion. When they are found in their glycosylated form, the absorption pathway is associated with active transporters, such as glucose transporter type 2 (GLUT-2), sodium-glucose cotransporter type 1 (SGLT-1), breast cancer resistance protein (BCRP), multidrug resistance proteins (MRP/P-gp), and sodium-coupled mono-carboxylate transporters (MCT) [25,26]. It has also been shown that the membrane-bound enzyme lactase-phlorizin hydrolase (LPH) is essential in the process of absorption of phenolic compounds and anthocyanins. LPH is a mammalian b-glycosidase found in the membrane brush border of the small intestine and has high affinity for several flavonoid glycosides [24,27].

In the small intestine, anthocyanins go through a process of conversion to lower-molecular-weight compounds, and although it is known that in the colon no absorption occurs, it is a crucial anatomical site, as it will be exposed to the intestinal microbiota, degrading them to secondary metabolites, impacting health. This biotransformation is considered phase II metabolism [28,29]. Anthocyanins and their conjugated metabolites are carried out of the enterocyte by efflux transporters, such as ATP Binding Cassette Subfamily C Member 2 (ABCC2) and MRPs/P-gp, then transported into the enterohepatic circulation, via the portal vein [30,31]. Once in the liver, flavonoids and other phenolic compounds are gradually hydroxylated and/or O-demethylated by cytochrome P450 (CYP450) and then undergo conjugation reactions (glucuronidation, sulfation, and O-methylation) catalyzed by phase II enzymes. Furthermore, flavonoids have been shown to be directly associated with the inhibition of cytochrome 2C9 (CYP2C9), and xenobiotic metabolism in the liver is closely coordinated with anthocyanin and phenolic compounds’ biotransformation [32,33,34]. Finally, these reactions produce more polar metabolites that will undergo one of the following pathways: (a) enter the general circulatory system to the kidneys for excretion in the urine, (b) transport to the tissues, and (c) return to the digestive system through the bile [19,35].

The characteristic C6-C3-C6 backbone is degraded to give rise to an extensive number of secondary metabolites, and this transformation will be conditioned by interactions with other nutrients, such as proteins, lipids, and some vitamins. It has been shown that the food matrix from which anthocyanins are consumed also determines their bioavailability [19]. Regarding this, anthocyanins have limited bioavailability—several studies have shown that only about 0.26–2% are absorbed intact [11,35]. Several authors agree that anthocyanin bioavailability is low, which creates a knowledge gap around these pigments, but also an area of opportunity for further research on their absorption, metabolism, and excretion [24,28].

### Gut Microbiota

Gut microbiota refers to a complex and diverse microorganism population that inhabits the host’s gastrointestinal tract. It is estimated that approximately 1000 species of bacteria coexist in the intestine. These bacterial populations exert multiple effects, having direct implications on health, for example, carbohydrate fermentation to generate energy, short-chain fatty acid (SCFA) formation, vitamin production (biotin and phylloquinone), and xenobiotics and phenolic compounds’ breakdown [36]. Within the microbiota heterogeneity, only certain species can metabolize phenolic compounds, including *Bifidobacterium* spp., *Lactobacillus* spp., *Bacteroides* spp., *Eubacterium* spp., *Gordonibacter* spp., and *Ellagibacter* spp. It has been shown that their presence is determined by interpersonal differences associated with factors such as dietary pattern, which plays a fundamental role in gut microbiota composition [37].

A substantial number of dietary polyphenols are not absorbed in the upper gastrointestinal tract; therefore, they encounter gut microbiota in the colon, biotransforming them into new phenolic compounds, which are absorbed and have beneficial effects on the different tissues. Dietary polyphenols are substrates for liver enzymes (phase I, hydrolyzing enzymes, and phase II, conjugating enzymes) and colon bacteria. Therefore, although the colon is not considered an absorption site per se, it is an active site for metabolizing phenolic compounds [38]. Gut microbiota plays a key role in the catabolism of phenolic compounds, and secondary metabolites produced by the microbiota are associated with bioefficacy in target organs. Bacteria mediate the formation of phenolic acids from polymers of larger phenolic compounds through ring fission and oxidation. It is noteworthy that colonic bioconversion of phenolic compounds is variable as a result of interindividual differences in gut microbiota [39]. Microbiota metabolism is also implicated in reactions, such as oxidation, demethylation, and breakdown of anthocyanins toward smaller phenolic compounds. The main metabolites of microbial degradation are protocatechuic acid, 4-hydroxybenzoic acid, vanillic acid, gentisic acid, phenylpropanoic acids (caffeic acid and ferulic acid), and hippuric acid [40,41].

Several studies show that some bacterial phyla can directly transform phenolic compounds. *Lactobacillus plantarum* efficiently metabolizes phenolic compounds by presenting glycoside hydrolases activity, and these enzymes catalyze the hydrolysis of glycosidic bonding of glycosylated phenolic compounds, including anthocyanins [42]. *Gordonibacter urolithinifaciens* and *Ellagibacter urolithinifaciens* are specialized bacteria that convert phenolic compounds, particularly ellagitannins and ellagic acid, into urolithins, which are health-promoting metabolites; besides, these bacteria can catabolize dietary phenols by cleaving hydroxyl groups from the catechol molecule and disrupt the double bonds [43,44]. *Bifidobacterium longum* is one of the most common species of the gut microbiota. It can specifically breakdown hydroxycinnamic acids (e.g., ferulic acid and p-coumaric acid) by its esterase activity, which selectively cleaves these compounds, leading to the production of metabolites, such as syringic acid and 4-hydroxybenzoic acid [45,46].

**Figure 2:**
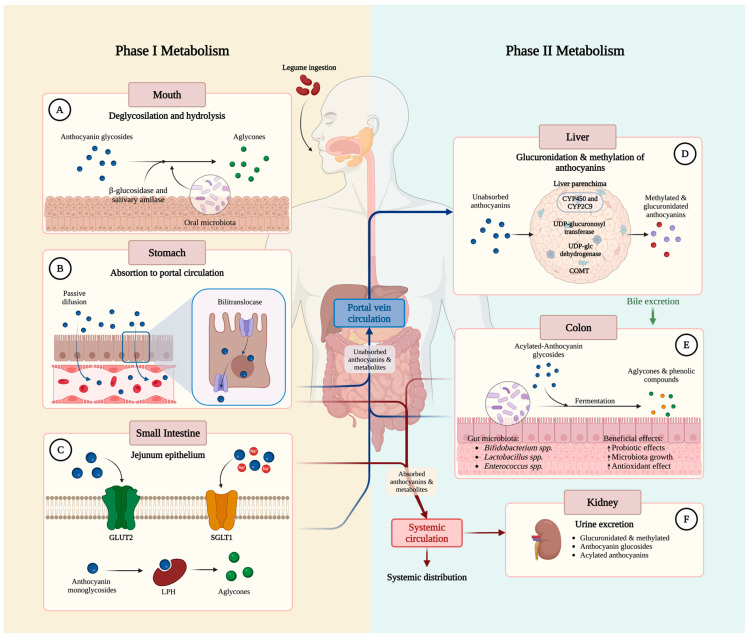
Phenolic compounds and anthocyanins’ metabolism: bioavailability, absorption, and excretion. Phase I metabolism. (**A**) The first step in the anthocyanins’ metabolism occurs in the mouth, where the anthocyanins are hydrolyzed by saliva enzymes, such as β-glucosidase and salivary amylase, and these enzymes eliminate the sugar conjugate, fabricating smaller and more bioavailable aglycones [28]. (**B**) As they arrive to the stomach, the acidic pH (1.5–3.5) confers more structural stability, increasing their bioavailability. Bilitranslocase is an organic anion carrier expressed in the gastric epithelium that serves as the main mechanism of absorption of anthocyanin glycosides in the stomach. (**C**) The small intestine is the major site of anthocyanins’ absorption (principally jejunum), and the primary role is that of the glucose transporter type 2 (GLUT2) and sodium-glucose cotransporter type 1 (SGLT1). Another mechanism of absorption in the jejunum is the degradation of anthocyanins in aglycones by the membrane-bound enzyme lactase-phlorizin hydrolase (LPH) [24]. The unabsorbed anthocyanin aglycones that come from the mouth can be absorbed either in the stomach and/or small intestine via passive diffusion [19]. Phase II metabolism. (**D**) Also, anthocyanins (including aglycones, glycosides, and acylated anthocyanins) from the stomach and small intestine enter to the portal circulation directly to the liver or stay in the intestinal epithelium to suffer phase II metabolism, where they primarily undergo oxidation and O-demethylated reactions by the cytochrome P450 and 2C9. Other phase II reactions occur in the liver and intestinal epithelium, such as conjugation with glucuronic acid via UDP-glucuronosyl transferase, methyl group via catechol-O-methyltransferase (COMT), and sulfate group via sulfotransferase and UDP-glc-dehydrogenase [16]. (**E**) Although there is no anthocyanin absorption in the colon, the intestinal microbiota plays a crucial role by converting acylated-anthocyanin glycosides into secondary metabolites, such as phenolic compounds and aglycones (bioactive substances with beneficial effects). (**F**) Once the anthocyanins are conjugated by phase II reactions in the liver, they have two destinations to be excreted from the organism. They first are delivered by systemic circulation to the target tissues (brain, liver, lungs, and kidneys) and, when they reach the kidney, anthocyanins are excreted in urine as glucuronidated and methylated aglycones (in the first 0–6 h after ingestion). In the second pathway, anthocyanins are distributed to bile, where they can be reabsorbed many times via enterohepatic circulation or undergo excretion through feces [24]. Created with BioRender.

Legumes are considered probiotic foods because they serve as bacteria food, increasing SCFA-producing species (*Bifidobacterium* spp., *Akkermansia* spp., and *Phascolarctobacterium* spp.) while decreasing negative ones, because of their potential pathogenicity and toxin production (*Staphylococcus* spp. and *Enterobacteriaceae* spp.) [47]. Moreover, their antibacterial activity has been demonstrated by decreasing overall bacterial diversity. Some of the proposed mechanisms are bacterial membrane degradation, nutrient deprivation, and toxin inhibition, to mention a few [48]. Interventions with phenolic compounds and anthocyanins have shown modifications in microbiota diversity; for example, consumption of 240 mg of anthocyanins per day/six weeks decreased firmicutes and proportionally increased bacteroidetes in mice fed with a diet high in anthocyanins but also in fiber [49]. These results suggest that a dietary intervention could promote eubiosis, which implies predominance of probiotic and SCFA-producing bacteria in relation to pathogens, and may have significant health outcomes, as the gut microbiota modulates physiological processes (Figure 2). The gut microbiota interacts with polyphenols in the following ways: (1) polyphenols modify the microbiota composition, (2) polyphenols influence microbial metabolism, and (3) microbiota transforms polyphenols in the colon. These complex interactions could explain the variability in the bioactivity, bioavailability, and absorption rate [50]. The interaction between the gut microbiota and phenolic compounds is complex and depends on multiple factors, so we recommend the following reviews for an in-depth understanding of the topic [51,52,53].

## 5. Results in Legumes

### 5.1. Beans

According to the Food and Agriculture Organization of the United Nations (FAO) information, beans (*Phaseolus vulgaris*) are the most widely consumed legume in the world, due to their high growth rate, easy environmental adaptation, and flexibility in seed size, color, and shape. In other words, beans can be a versatile food, easily accessible and well accepted in several regions of the world [54]. Beans are a source of bioactive compounds, such as fermentable non-digestible components, resistant starches, oligosaccharides, and phenolic compounds. The latter exert antioxidant and anti-inflammatory effects and have potential applications in metabolic disease treatment (T2DM, nonalcoholic fatty liver disease, and metabolic syndrome) [55,56,57]. The coat contains the highest concentration of phenolic compounds, including catechins, tannins, saponins, chlorogenic acid, ferulic acid, gallic acid, and anthocyanins, and these components are associated with cell damage protection and metabolic parameters’ improvement [57,58]. Additionally, it has been seen when making the translation of the phenolic compounds to other types of food, such as rice, that there is an importance in the amount of water used, as this has a direct impact on the phenolic concentration [59].

#### 5.1.1. Anti-Inflammatory and Antioxidant Protective Effect Against Cell Damage

Red bean (*Phaseolus radiatus* L. *var. Aurea*) is a legume native to Asia. It is recognized in traditional medicine for its antioxidant effect, and it is now known that anthocyanins are partially responsible for both its color and its effect, where delphinidin-3-glucoside (D3G), cyanidin-3-glucoside (C3G), and peonidin-3-glucoside (P3G) exhibit the highest concentrations. In addition, in a study on macrophages, it was shown that a lyophilized red bean inhibited nitrite production in a lipopolysaccharide (LPS)-induced, dose-dependent manner. On the other hand, red bean treatment significantly decreased the formation of ROS, such as hydrogen peroxide (H_2_O_2_). LPS initiates the proinflammatory cascade, promoting the secretion of proinflammatory cytokines, which activates the NF-κB, activating the expression of proinflammatory genes. Nevertheless, the excessive formation of ROS induces oxidative stress, resulting in irreparable cell damage associated with disease, so the red bean extract could help reduce inflammation and oxidative stress [58].

A navy bean (white) and black bean diet was tested in an in vivo colitis model with C57BL/6 mice, where a decrease in circulating levels of inflammatory cytokines, interleukin 1β (IL-1β), TNF-α, interferon-gamma (IFN-γ), and interleukin 17 (IL-17A), was observed; likewise, it enhanced interleukin 10 (IL-10), which is considered an immunoregulatory cytokine. Regarding colonic mRNA expression, both bean diets decreased *Il-6*, *Ifn-γ*, and *Il-22*, and increased *Il-10* in a significant manner. In addition, an elevation in SCFA was also shown in the cecum—butyrate, propionate, and acetate concentrations were increased in the bean-consuming groups compared to the control. Furthermore, evidence supports that the nutritional qualities of beans modulate colonic inflammation, due to fermentable carbohydrates and phenolic compounds, besides contributing to microbiota eubiosis by increasing SCFA-producing species, which indirectly improves the inflammatory state associated with colitis disease. On the other hand, histological damage in the colon increased with either diet consumed, being significant when consuming navy bean [57].

#### 5.1.2. Lipid-Lowering and Prebiotic Effect

Another variety of red bean, known as Adzuki bean (*Phaseolus angularis*), was tested in a trial in C57BL/6 male mice. Adzuki bean is known to be a source of anthocyanins, catechins, and adzukisaponins, and these bioactive compounds may influence the regulation of hepatic lipid accumulation. After 10 weeks, body weight was reduced, along with decreased hepatic triglycerides (TG), total cholesterol (TC), low-density lipoprotein (LDL), and very-low-density lipoprotein (VLDL) cholesterol levels, and proportionally increased high-density lipoprotein (HDL) cholesterol levels compared to the control groups that received either a normal or a high-fat diet (HFD). In this sense, the expression of lipogenic genes (*Srebp-1c*, *Cpt-1*, and *Fas*) was downregulated and adiponectin (*Adipoq*) increased comparing both bean groups to the HFD group. Although, a difference was seen between groups, as proliferator-activated receptor alpha (PPAR-α) increased only in one group. When evaluating the antioxidant enzyme capacity, superoxide dismutase (SOD), glutathione peroxidase (GPx), and catalase (CAT) increased, and there was a decrease in mRNA expressions of *Tnf* and *Nf-κb* in both bean groups. Therefore, it was evidenced that Adzuki beans not only lowered hepatic lipid accumulation but also decreased inflammation in a murine model [56].

Likewise, in a study conducted in Wistar-Kyoto rats and spontaneously hypertensive rats, the Adzuki and black bean extracts showed a beneficial impact on TG, TC, and systolic blood pressure. In addition, they reduced relative liver weight, liver enzymes, and angiotensin-converting enzyme levels compared to controls. The angiotensin II level in the Adzuki group and *renin* mRNA expression in both groups were significantly lower compared to the control [60]. This information gives an idea of the health benefits associated with bean consumption; in synergy, their bioactive compounds, including flavonoids, terpenes, alkaloids, and phenolic acids, could have an effect that improves the lipid profile, in both serum and liver. Additionally, it regulates the expression of genes related to lipid metabolism, as well as antioxidant and anti-inflammatory capacity.

#### 5.1.3. Anti-Obesity and/or Antidiabetic Effects

The black bean (*Phaseolus vulgaris* L.) is characteristic of America and has taken relevance due to its intense color. In the husk are concentrated different types of anthocyanins: petunidin-3-O-glucoside (Pe3G) represents the highest proportion (56%), followed by D3G (34%) and M3G (10%). It was demonstrated that this anthocyanins profile, typical of a Mexican variety, inhibited enzymes such as α-glucosidase (37.8%), α-amylase (35.6%), dipeptidyl peptidase-IV (34.4%), ROS (81.6%), and decreased glucose uptake in an enzymatic in vitro study (test tubes). Regarding anthocyanins’ inhibitory activity, they were inferior compared to the controls used. Acarbose showed a higher enzymatic inhibition toward α-glucosidase and α-amylase (60.9% and 66.8%, respectively), and sitagliptin presented a 99.6% enzymatic inhibition of dipeptidyl peptidase-IV. This study illustrated the anti-obesity and antidiabetic potential of black bean anthocyanins through an insulin-independent mechanism, simulating inhibition of glucose uptake in the intestine [61].

Similar results were obtained when evaluating the effect of black bean in Wistar diabetic rats: after five weeks of treatment, fasting blood glucose and TNF-α levels decreased in the black bean group. On the other hand, a differential expression of miRNAs (miR-152, miR-219a1, and miR-384) was observed between the bean group and the control, and these miRNAs are particularly important because are altered in diseases such as type 2 diabetes mellitus (T2DM) and obesity. Bioinformatic analysis had indicated that black bean anthocyanins interact with proteins, such as insulin-1 precursor (INS1) or inositol polyphosphate phosphatase-like 1 (INPPL1), and produce changes in their activity, which modulates their downstream activity. In addition, a molecular docking assay highlighted that anthocyanin conjugates have a high affinity for certain transcription factors, such as D3G, Pe3G, and M3G to GATA2 and POU2AF1. These changes could explain the bean’s regulatory effects, since these transcription factors play an important role in adipose tissue and the immune system, as well in the evolution and progression of metabolic diseases, such as T2DM [62].

Furthermore, Sun et al. showed that the black bean (and black rice) decreased glucose intolerance, insulin levels, and homeostatic model assessment for insulin resistance (IR) in diabetic rats. Compared with the control group, in the black bean group, TNF-α and IL-1β significantly decreased, whereas IL-6 and IL-10 significantly increased in the treatment group. Likewise, after treatment, oxidative stress status decreased, as evidenced by an increase in liver malondialdehyde (MDA) and endogenous antioxidant response enzymes, such as SOD and CAT. Besides, the lipid profile improved, suggesting that the antioxidant and anti-inflammatory capacity of black beans influences lipid metabolism and the inflammatory status in T2DM [63].

A crossover clinical trial in subjects with metabolic syndrome (MS) showed that including black beans in a meal had positive effects on the postprandial insulin peak compared to diets similar in fiber and antioxidant capacity. The above indicates that a simple dietary modification could improve health parameters involved in MS [55]. Recently, another crossover clinical trial, performed in healthy subjects, compared the postprandial glucose and insulin response after consumption of black beans and white bread. Three hours later, the incremental area under the curve (iAUC) of glucose and insulin decreased significantly in the group that consumed 100% black bean pasta, in relation to the group that consumed white bread. An important finding indicated that black bean consumption increased satiety and decreased appetite. This demonstrates that the type of carbohydrate consumed can modulate metabolic responses [64].

Many of the beneficial effects of beans, and in general legumes, could be attributed to polyphenols, flavonoids, and hydroxycinnamic acid. These molecules play a protective role against environmental damage to plants and, when consumed, improve health status by regulating molecular pathways, such as nuclear factor erythroid 2-related factor 2 (Nrf2), adenosine-monophosphate-activated protein kinase (AMPK), and mitogen-activated protein kinase (MAPK), to mention some [56,63,65,66,67]. However, the concentration of these compounds, also known as phytochemicals, is variable and depends on environmental factors. For example, Salas-Lumbreras et al. conducted a comparative study of the same variety of bean (Flor de Junio Dalia) with different levels of water restriction during growth in an obesity Wistar rat model. At the end of the study, all groups that consumed beans showed weight loss and improved the lipid profile compared to the group consuming the HFD. Finally, an inhibition of α-glucosidase was higher in water-deprived seeds compared to fully irrigated ones [68].

Results indicated that water-deprived seeds had higher concentrations of dietary fiber and phytochemicals, including phenolic compounds, flavonoids, and saponins. It has been shown that water restriction generates stress in the plant, which increases the production of phytochemicals to counteract ROS generation. Maintaining a deficit in water irrigation could be an agronomical cultivation practice to improve the functional profile of bioactive compounds in legumes [68,69].

#### 5.1.4. Other Health Effects

Skin anti-aging effects of black bean have been explored. In an enzyme inhibition study (in vitro, test tubes), inhibition of tyrosinase and elastase activity was observed, with enzymes associated with hyperpigmentation and skin elasticity, which may have a potential function for the cosmetic industry. Also, there was a clear emphasis on the method of purification, as this is essential to provide a greater antioxidant capacity [70].

#### 5.1.5. Conclusion

It is proposed that beans improve oxidative stress and inflammation through several molecular pathways: on the one hand, by decreasing circulating inflammatory cytokines and downregulating their gene expression, and on the other by inhibiting pathogen-associated pathways, e.g., one induced by LPS, activating proinflammatory cascades, is NF-κB. LPS reduction is also associated with gut microbiota homeostasis, which produces SCFA and diminishes the pro-oxidant and inflammatory state, in in vitro assays. Based on their unique phytochemical profile, beans promote nutrigenomic modifications that mitigate inflammation and oxidative stress. This is a promising therapeutic approach in a wide range of diseases.

Likewise, in vitro and in vivo studies have shown that anthocyanins and phenolic compounds, present in beans, can inhibit key enzymes related to carbohydrate digestion, improve the lipid profile, and decrease inflammation markers and oxidative stress, highlighting their potential in the prevention and treatment of metabolic diseases. In addition, as an innovative approach, the anti-aging effect has been explored (Table 1).

In human studies, black bean consumption showed positive effects on postprandial glucose and insulin response, increasing satiety and decreasing appetite, suggesting that dietary modifications can have a significant impact on metabolic health. This evidence underlines the importance of continuing to evaluate the health effects of beans in human.

Besides anthocyanins and phenolic compounds, several bioactive compounds can be found in the bean, including saponins, terpenes, and alkaloids, which in synergy contribute to its health effects.

### 5.2. Soybean

Soybean (*Glycine max*) is an important legume that provides protein and oils for human consumption [71]. There are different soybean varieties, and their anthocyanin content is merely distributed in black soybeans. The pigment is localized in the seed coat and is especially abundant in C3G [9,72,73]. The anthocyanins from this variety are more stable than other plants, such as strawberries, and provide plant UV damage protection and resistance to pests [71,73]. The black soybean is native to Asia, where it has been consumed as a health-promoting food and has been investigated to prove these benefits in both in vitro and in vivo studies [67,74].

#### 5.2.1. Anti-Obesity Effect

In vitro and in vivo studies have addressed the effect of black soybean extract (BSE) on different aspects of obesity pathophysiology. The treatment with black soybean reduced the size of adipocytes, improved glucose uptake and adiponectin production, and upregulated *Pparγ* and *Cebpa*. On the contrary, it decreased the production of TNF-α in 3T3-L1 adipocytes. In line with these results, BSE prevented body weight gain and decreased food consumption in mice in the same study [75].

BSE has been shown to positively affect energy metabolism and thermogenesis. BSE decreased TG accumulation in adipocytes and increased uncoupling protein 1 (UCP1), peroxisome proliferator-activated receptor γ coactivator 1 α (PGC1-α), and cytochrome c proteins in mice. These findings suggest BSE induces mitochondrial activation energy metabolism, which could be targets to treat obesity [66].

Wu et al. investigated oxidative stress and inflammation in diet-induced obesity in mice. After 12 weeks of treatment with BSE, the body weight decreased by 13.3%, which may suggest a decrease in the food utility rate. Additionally, the lipid profile and expression levels of *Tnf*, *Il-6*, *Nf-κb*, and *iNOS* genes decreased, and total SOD and GPx activities significantly increased, indicating that BSE could partially prevent diet-induced oxidative stress and liver inflammation [76].

The last investigation into obesity bore the effect of a black soybean on neuroendocrine regulation of appetite, which has been linked to obesity. After treatment, the weight gain and food consumption per day were reduced. Downregulation of neuropeptide Y (NPY) and γ-amino butyric acid receptor (GABAB1R) in the hypothalamus was also observed. The anti-obesity effect could partially be explained by the decreased appetite and food consumption regulated via NPY and GABAB1R [77].

#### 5.2.2. Antidiabetic Effect

The antidiabetic effect of BSE has been studied with the hypothesis of ameliorating obesity-associated IR by alleviating inflammatory changes and adipocyte dysfunction in 3T3-L1 adipocytes co-cultured with macrophages. The BSE generates a significant suppression of ROS production. ROS are produced when there is excessive fat accumulation and are related to the dysregulated production of inflammatory mediators and cytokines. The treatment with BSE inhibited the increase of nitric oxide (NO), monocyte chemoattractant protein 1 (MCP-1), prostaglandin E2 (PGE2), TNF-α, and IL-6, and reduced the migratory ability of macrophages. When adipose tissue is enlarged, it increases the release of free fatty acids (FFA) and the secretion of inflammatory cytokines, such as MCP-1. This is associated with the activation of NF-κB and MAPK pathways, including extracellular signal-regulated kinase (ERK) and c-Jun N-terminal kinase (JNK). Pretreatment with BSE inhibited the LPS-induced phosphorylation of ERK and JNK. FFA, MCP-1, and TNF-α enhance adipose tissue’s inflammatory responses and IR. In this study, the adipocytes co-cultured with macrophages failed to increase glucose uptake in the insulin response, indicating IR induction. However, treatment with BSE increased the glucose uptake by 45%. To identify a possible mechanism, they investigated PPAR-γ, a transcription factor regulating the expression of genes associated with adipocyte function. The treatment of co-cultured cells restored PPAR-γ protein expression, causing a 91% increase [67].

Studies have addressed the problem from different perspectives in the same research. In the HepG2 cell line, which mimics the lipid disorders seen in T2DM, the pretreatment with BSE significantly increased CAT, SOD, and GPx, decreased the MDA content, and exhibited an inhibitory activity on α-amylase. Similar results were observed in induced insulin-resistant diabetic mice in this study, where it also lowered fasting blood glucose, blood glucose tolerance, insulin levels, and HOMA-IR [72].

Yamashita et al. investigated the impact of a single dose of procyanidin oligomers (procyanidin B2, procyanidin C1, cinnamtannin A2, and structural isomer of tetramer EC-(4β–6)-EC-(4β–8)-EC-(4β–8)-EC, PA2) isolated from black soybean over glucose metabolism. After 60 min of a single dose of 10 μg/kg, the procyanidins activated insulin and AMPK signaling pathways to induce GLUT4 translocation in muscle and suppress acute hyperglycemia in mice. Cinnamtannin A2 showed the strongest effect. The perspective presented is that a daily intake of 10 g per person of black soybeans is an appropriate quantity to see significant clinical results [78].

#### 5.2.3. Lipid-Lowering Effect

BSE mitigated hyperglycemia while improving antioxidant enzyme activity and had benefits in hyperlipidemia. An investigation studied the effects of both metabolic alterations and, additionally, the antidiabetic effect discussed before. They observed a decrease in lipid accumulation in the HepG2 cell line and lowered serum FFA, TG, and TC while increasing HDL in induced insulin-resistant diabetic mice [72].

It is common for studies to use more than one intervention. This occurred in a study where BSE and bean extract were administered to different groups of mice. Similar results were seen on lipid metabolism, but the effects showed stronger evidence in beans. It is necessary to mention the focus of this study was the antihypertensive effect, where they found positive results [60].

Diabetic nephropathy is a medically important complication of T2DM, and it is associated with oxidative stress and lipotoxicity. In this sense, a link between AMPK inhibition and lipotoxicity has been reported, which could be related to inflammation, oxidative stress, and subsequently, diabetic nephropathy. On the other hand, anthocyanins are emerging as AMPK activators. In addition, PPARs transcription factors are noteworthy as being of biological importance because they can regulate key metabolic pathways involved with lipid metabolism. Koh et al., in an in vivo study (diabetic mice), indicated that BSE decreased TG, TC, and albuminuria. It also improved inflammation markers and oxidative stress related to nephropathy, such as transforming growth factor beta 1 (TGF-β1) and 8-epi-prostaglandin F2α. On the other hand, it minimized kidney lipid deposition through AMPK activation and upregulation of the transcription factors PPAR-α and PPAR-γ, which mediated lipid accumulation in the kidney [79].

Only one clinical trial has been performed with BSE alone: an 8-week intervention providing BSE to participants between 19 and 65 years. Overweight or obese participants reported a decrease in weight, waist circumference, and body mass index (BMI); however, there were no significant differences compared to the control group. Lipid profile markers LDL, non-HDL, and LDL/HDL showed improvement. This indicates that the anthocyanins from BSE may be a promising strategy in the treatment of obesity, although there are still important aspects to be defined, such as the optimal time and dosage or quantity for human consumption. Furthermore, it will be relevant to study the molecular mechanisms underlying these results [74].

#### 5.2.4. Other Health Effects

In our search, we found that BSE may have other positive effects on health. The evidence suggests BSE may potentially be used to prevent cataracts. In the HLE-B3 cell line, BSE treatment significantly decreased BAX and BAD, pro-apoptotic members of the BCL2 protein family (with increased BCL2), p53, a tumor suppressor, and caspase-3, a protease which is associated with apoptosis [80].

Studies with in vitro and in vivo experiments showed similar results in BCL2. In DU-145 cells, BSE intervention caused a significant increase in apoptosis and decrease in BCL2, PSA, and AR. In mice, treatment significantly suppressed tumor growth. With these results, it was proposed that BSE can not only suppress but also inhibit prostate cancer progression [81].

BSE could improve the wound healing process. Treatment with BSE exerted antioxidant and cytoprotective effects by protecting key cells involved in wound healing (keratinocytes and fibroblasts) from oxidative stress induced by ROS, thereby increasing their viability and function under damage conditions. This cytoprotective effect was accompanied by the modulation of inflammation, inhibiting the translocation of the transcription factor NF-κB to the nucleus and reducing IκBα phosphorylation in rat wounds. These antioxidant and anti-inflammatory actions are interrelated with BSE’s ability to enhance angiogenesis by increasing VEGF and decreasing TSP1 levels and promoting an organized deposition of collagen [82].

The antimicrobial effect was seen with a significant reduction in prostatic inflammation using BSE in rats with chronic bacterial prostatitis [83]. Also, neuroprotective activities on neurological disorders in mice have been observed. The mice were injected with LPS (250 μg/kg) for 7 days and BSE (24 mg/kg) for 14 days. The authors found a significant decrease in inflammatory mediators, such as TNF-α, IL-1β, and COX-2, in the cortex. The same results were observed when measuring the oxidative stress and the effects over ROS. These results suggest that the treatment would decrease LPS-induced ROS and oxidative stress in neuroinflammation [84] (Table 2).

#### 5.2.5. Conclusion

Soybean anthocyanin has a significant antioxidant and anti-inflammatory effect by suppressing ROS production in in vitro models. ROS production is related to the dysregulated production of inflammatory mediators and cytokines in excessive fat accumulation. Besides, it inhibits LPS-induced phosphorylation of the ERK and JNK pathways, both of which are involved in the inflammatory response. Moreover, the antioxidant capacity of soy phenolic and anthocyanin compounds has a far-reaching application and has been revealed through their neuroprotective effects, antimicrobial activity, and accelerated skin wound healing.

The anti-obesity effect of soybean may encompass its inflammation-regulating effect, but it is important to emphasize that other in vivo studies have evaluated other important components of obesity, such as neuroendocrine regulation of appetite and thermogenesis.

Soybean has several antidiabetic effects that have been demonstrated both in vitro and in vivo. Black soybean anthocyanins increased glucose uptake in adipocytes co-cultured with macrophages, indicating an improvement in insulin sensitivity. On the other hand, procyanidin oligomers, isolated from black soybean, activate insulin and AMPK signaling pathways, promoting GLUT4 translocation in muscle and suppressing acute hyperglycemia. It has also been found to decrease serum lipid levels, avoid lipotoxicity in the liver, and upregulate the transcription factors PPAR-α and PPAR-γ. These effects combined suggest that soybean potentially improves glucose and lipid metabolism, reduces oxidative stress and inflammation, and mitigates complications associated with T2DM, such as diabetic nephropathy. Importantly, most of the studies reported were in vivo and in vitro, and only one human clinical trial was documented, which revealed a positive effect of soybeans on body weight and anthropometric measurements, so it is imperative to continue undertaking research and performing the translation of the findings to clinical trials.

### 5.3. Lentils

Lentils (*Lens culinaris*) are named for their round and curved shape, resembling a lens. Nutritionally, they are high in dietary fiber, protein, vitamins, minerals, and antioxidant compounds such as phenolic compounds, flavonols, anthocyanins, tannins, and saponins. Based on their nutritional content, lentils are considered superfoods, as they combine various essential nutrients and bioactive compounds. They are also considered cost-effective legumes due to their high nutritional value, low cost, environmental sustainability, and consumption versatility. There is a wide variety of characteristic colors, such as brown, yellow, orange, red, and black lentils, according to where they were cultivated and the type of bioactive compounds present in the coat. Currently, it has been demonstrated that their nutritional profile has a key role in the treatment and prevention of diseases, such as T2DM, obesity, and cardiovascular diseases. The following sections summarize the up-to-date information on the health effects of lentils [85,86].

#### 5.3.1. Anti-Inflammatory and Antioxidant Protective Effect Against Cell Damage

Beluga lentils stand out from other varieties due to their small size and intense black color, which is provided by a high concentration of anthocyanins. In a study conducted by Jung et al., they evaluated the antioxidant effect of beluga lentils in a cellular and murine model, inducing hepatotoxicity. Bioactive compounds from lentils were extracted with organic solvents and subsequently lyophilized. The results showed that beluga lentil extract significantly decreased ROS in H_2_O_2_-treated AML12 cells (mouse hepatocytes), which showed the direct ROS scavenging activity of beluga lentils. On the other hand, it was observed that pretreatment with 100 μg/mL of beluga lentils for six hours promoted the activation of the transcription factor Nrf2, which would partially explain the antioxidant mechanism. In relation to the murine model, pretreatment with 400 mg/kg of beluga lentils for two weeks significantly reduced serum ALT and AST levels, enzymes elevated in physiological processes of hepatotoxicity. Regarding the antioxidant activity, the group that consumed the lentil extract presented an upregulation in the expression of endogenous antioxidant pathway genes, such as *Gpx2* and *Sod1*, mediated by Nrf2, which is in accordance with the findings in the in vivo model [65].

#### 5.3.2. Lipid-Lowering and Prebiotic Effects

Lentils are a good source of soluble and insoluble fiber. These types of carbohydrates are not digested in the upper gastrointestinal tract and reach the colon practically intact, where they are digested and metabolized by the intestinal microbiota bacteria, which is why they are known as prebiotics. On the other hand, several studies have shown that bioactive compounds in legumes could improve parameters related to cardiovascular risk, such as the lipid profile. Micioni Di Bonaventura et al. analyzed the lipid-lowering and prebiotic effects of a lyophilized extract derived from lentils in a murine model. The results indicated that the treatment group significantly decreased TC levels compared to the control group, accompanied by an increase in HDL cholesterol and a decrease in LDL cholesterol [47].

Concerning prebiotic potential, lentils increased the colony-forming units of *Bifidobacterium* spp., bacteria considered beneficial, and decreased their negative counterparts, *Staphylococcus* spp. and *Enterobacteriaceae* spp. In addition, SCFA, produced by the microbiota after prebiotic fermentation, increased in the lentil group, especially butyric acid. Furthermore, it was shown that lentils’ saponins (components of soluble fiber) significantly lowered TC [47]. Dissimilar results were obtained in an in vitro assay focused on colonic fermentation: lentil saponin-rich extract decreased the growth of bacteria, for example, *Enterococcus* spp., *Clostridium* spp., *Bifidobacterium* spp., and *Lactobacillus* spp. This study demonstrated the widespread antibacterial capacity of lentils, decreasing damaging species as well as those with probiotic effects (*Bifidobacterium* spp. and *Lactobacillus* spp.). Regarding this, it is important to mention that reducing the bacterial population could contribute to an eubiosis state, decreasing bacteria overgrowth and potentially compromising homeostasis; furthermore, it was evidenced that the transformation of lentil saponins to sapogenins by the human intestinal microbiota exhibited a modulatory effect on the growth of selected intestinal bacteria [48]. The discrepancies in the results could be due to the difference in the study model, the lentil variety, and the extraction method; nevertheless, in both studies, it was possible to identify the close relationship between lentils and the gut microbiota.

Lentils have also shown beneficial effects in humans. According to the results reported by Aslani et al., conducted in subjects with obesity and diagnosis of T2DM, it was evidenced that eight weeks of consumption of lentil sprouts decreased the levels of TG and oxidized LDL cholesterol (ox-LDL); in contrast, it increased HDL cholesterol levels compared to the control group. Another important finding was a decrease in the TG/HDL-c ratio, which is considered an important indicator of cardiovascular risk [87].

#### 5.3.3. Anti-Obesity and Antidiabetic Effects

Carbohydrates in lentils are composed of slowly digestible starch, rapidly digestible starch, and resistant starch fractions. They also have a high protein content, which contributes to the low glycemic index (GI) and available carbohydrates (AC) found in food. Results from a human trial carried out by Ramdath et al. confirmed that lentils are a low-GI food because consuming them decreases the postprandial glucose response. They were shown to have intact cellular structures after cooking, which maintains the dietary fiber content and anti-nutrients (e.g., phenolic compounds). This crossover clinical trial was compared between white bread and cooked lentils (25 g of AC) and demonstrated that none of the lentil examples produced a glucose spike, in contrast to white bread, where this became more evident after 45 min of consumption [88].

There is evidence that phenolic compounds may influence starch digestion. It seems that phenols form complexes with the amylose fraction of starch, which increases resistant starch—this phenol–starch conjugate could explain the prebiotic effect of legumes [88,89]. Based on this, Moravek et al. conducted a comparative study between two high-GI foods (rice and potato) and one low-GI food (lentils), as well as a blend of both (50% of AC of potato and rice was replaced by lentils). Findings from this crossover trial, conducted in overweight or obese patients, showed that compared to rice and potato alone, lentils (single or mixed) significantly lowered postprandial blood glucose iAUC and maximum concentration (Cmax) at 15, 30, 45, and 90 min following consumption. Components of lentils affect and slow down starch digestion, reducing the postprandial glucose response, including polyphenols that affect α-glucosidase activity and form complexes with starch, slowing its absorption. Replacing half of the AC in high-starch foods with legumes could be a promising nutritional strategy in T2DM treatment [90].

Accordingly, it has been shown that consuming lentils as part of the regular diet has beneficial effects by avoiding an increase in the HOMA-IR index, which is considered an important indicator of the degree of IR. Legumes can improve the IR profile and are a key factor in the progression of T2DM. Their high fiber content delays gastric emptying as well as the absorption of AC, showing benefits in the acute postprandial glucose and insulin response [91].

#### 5.3.4. Other Health Effects

Black lentil anthocyanins may have a beneficial effect on cancer by regulating specific molecular pathways. Mazewski et al. demonstrated that black lentils showed greater effectiveness in inhibiting colon cancer cells (62.2% inhibition) in cell culture than other foods, such as rice, sorghum, or grapes. The most common anthocyanin in the lentil coat was D3G, whose metabolites could directly contribute to ameliorating the inflammatory state. Anthocyanins have been identified to regulate cancer cell apoptosis through their interaction with inhibitory apoptosis proteins, as well as modulate angiogenesis by regulating growth and proliferation factors, such as vascular endothelial growth factor receptor 2 (VEGFR2) and epidermal growth factor receptor (EGFR), which was evidenced in an in silico analysis. C3G, D3G, P3G, and Pe3G showed the lowest IC30 in inhibiting the growth of the colon cancer cell line. Additionally, black lentil extract was the most potent of the plant extracts in terms of IC50 for HCT 116 cells [92] (Table 3).

#### 5.3.5. Conclusion

There is a wide variety of bioactive compounds or phytochemicals in lentils (e.g., phenolic compounds, anthocyanins, saponins, etc.), collectively attributed to having a beneficial effect on health. Lentil phytochemicals exert their antioxidant activity by activating Nrf2, which works as a transcriptional trigger of endogenous antioxidant response genes, e.g., *SOD*, *CAT*, and *GPx*, which encode for enzymes that inhibit ROS and, therefore, oxidative stress and inflammation. Besides, fiber is a major component of lentils and legumes in general. This fiber has a prebiotic function, as it is fermented by the gut microbiota, enhancing the SCFA-producing bacteria, which translates directly into an enhancement of intestinal health and indirectly into an improvement of metabolic health. This information needs to be translated to clinical trials to specifically explore lentil health effects and develop recommendations.

Diverse clinical trials have examined lentils’ effect on glucose metabolism, considering their profile of carbohydrates, phytochemicals, and proteins that, in combination, contribute to a prolonged satiety sensation, delaying gastric emptying and absorption of available carbohydrates. This may help control appetite, reduce total caloric intake, and improve glycemic control. Specifically, the polyphenols in lentils affect α-glucosidase activity and form complexes with starch, which slows its absorption and reduces the postprandial glucose response. In addition, black lentils have exhibited a promising therapeutic effect by modulating molecular biomarkers associated with cancer and its progression.

### 5.4. Chickpeas and Legume Mix

One of legumes’ most important nutritional aspects is that they can lower postprandial glucose due to their fiber and slow-digestion starch content. Chickpeas have been studied regarding to this effect. A study by Winham et al. indicated that consuming chickpeas in a mixture with rice significantly lowered glucose levels compared to rice alone 60 and 90 min after consumption [93]. Mixing legumes with cereals has been the basis of the diet of many cultures; in addition to improving their nutritional value, the health benefits are now known and can be applied in the nutritional approach to diseases. Similar results were observed by Bajka et al. when replacing 30% or 60% of wheat flour with chickpea flour in bread. A positive effect on postprandial glucose responses was observed—by adding 60% chickpea flour, postprandial glucose decreased compared to wheat bread [94]. Both studies highlighted the positive effects of legume consumption, particularly promoting satiety. Bajka et al. demonstrated that incorporating 60% legume cellular powder into bread significantly increased GLP-1 and PYY responses, offering a potential dietary strategy to enhance satiety. Similarly, Winham et al. discovered that legumes increase satiety by releasing CCK and GLP-1, indicating that legumes are a valuable protein source for appetite regulation.

According to some studies, the consumption of legumes (mixed pulses) has been shown to improve health parameters: it lowers TC levels, improves blood pressure [95], and has a positive effect on the postprandial glycemic response [96] (Table 4).

#### Conclusion

In summary, a variety of pulses in our daily life offer profound health advantages. Research highlighted that consumption of legumes demonstrated significant effects in reducing the glycemic response when incorporated in the diet, demonstrating their efficacy in managing postprandial glucose and insulin levels.

Amidst the growing incidence of chronic diseases and obesity, discovering pertinent dietary strategies is vital. Introducing innovative legume-based ingredients into food, such as chickpea consumption, which has been shown to reduce postprandial glucose levels in articles included in this review, exemplifies the beneficial effects on glycemia, insulinemia, and satiety hormone release. On the other hand, some authors reported a decrease in the lipid profile and decreased appetite by having an impact on the release of anorexigenic or intestinal hormones.

Overall, integrating legumes into the diet can enhance nutritional quality and contribute to a healthy, balanced lifestyle, notably by lowering circulating cholesterol and glucose levels, thereby reducing the risk of metabolic and cardiovascular diseases.

**Figure 3:**
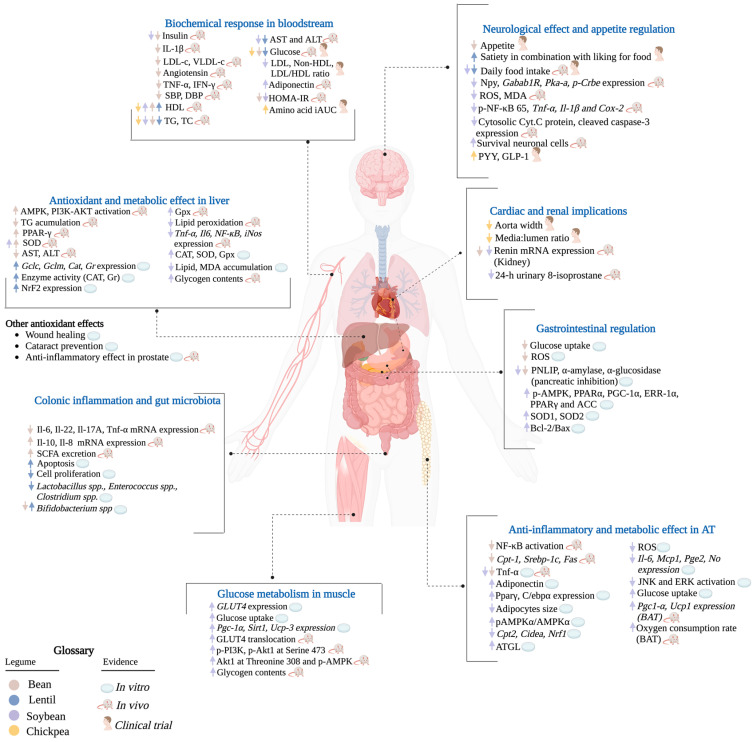
A broad overview of legumes’ effects in inflammation, oxidative stress, and metabolism, by anatomic site. As reviewed, the effects of anthocyanins take part in many organs and tissues, involving anti-inflammatory pathways, glucose, carbohydrate, and lipid metabolism, and regulation of neuro-hormonal processes, such as appetite, and gastrointestinal, cardiac, and renal function. Most results were obtained from in vivo and in vitro studies, highlighting bean, lentil, and soybean, the three most consumed legumes worldwide. The main results are focused on anti-inflammatory and antioxidant effects, lipid-lowering properties, and glucose metabolism. However, legumes improve other metabolic responses, such as the gut microbiota environment, colonic health, inflammation, and wound healing. From this perspective, legumes have a particular benefit in cardiovascular health (with a clear focus on cardiovascular risk assessment) regarding the biochemical response in the bloodstream, with the reduction of cardiovascular risk biomarkers, for example, low-density lipoprotein cholesterol (LDL-c), very-low-density lipoprotein cholesterol (VLDL-c), triglycerides, total cholesterol, systolic and diastolic blood pressure (decreased renin and angiotensin expression), and glucose, and elevation of high-density lipoprotein (HDL-c). Neuroprotective effects have also been shown due to reduced anti-inflammatory cytokines: interleukin-β (IL-β), tumor necrosis factor-α (TNF-α), nuclear factor-kB (NF-kB), and prostaglandin-endoperoxide synthase 2 (COX-2), in the cortex. Likewise, phenolic compounds and anthocyanins reduced oxidative stress in neuronal tissue by diminishing reactive oxygen species (ROS) and malondialdehyde (MDA). There was evidence of a neuroendocrine regulation of anti-obesity mechanisms and appetite regulation, mainly a downregulation of neuropeptide Y (NPY) and γ-amino butyric acid receptor (GABA1R). Another important activity, associated with phenolic compounds and anthocyanin metabolism, is the probiotic effect and gut microbiota regulation. This is accomplished through the variety of reactions performed by the bacteria in the colon, converting anthocyanins into phenolic compounds and secondary metabolites when short-chain fatty acid (SCFA) formation increases. This causes a regulation in the bacteria population, decreasing negative ones, such as *Staphylococcus* spp. and *Enterobacteriaceae* spp., and increasing SCFA-producing bacteria, such as *Bifidobacterium* spp., *Akkermansia* spp., and *Phascolarctobacterium* spp., thus achieving eubiosis in the gut microbiota. In general, anthocyanins work as potent antioxidants by decreasing oxidative stress in the liver, enhancing some enzymes, such as catalase (CAT), superoxide dismutase (SOD), and glutathione peroxidase (GPx), and decreasing MDA accumulation in hepatocytes. The lipid-lowering action is achieved by upregulating enzymes such as peroxisome proliferator-activated receptor gamma (PPAR-γ), causing a decrease in lipid accumulation and taking part in the antidiabetic effects of anthocyanins, causing a reduction in insulin resistance, insulin plasmatic levels, fasting glucose plasmatic levels, and homeostatic model assessment for insulin resistance (HOMA-IR). Finally, anthocyanin effects in obesity and diabetes models have shown great benefits in the downregulation of adipose tissue inflammation biomarkers and adipose hormones, an improvement in glucose metabolism in muscle, and the regulation of digestion and the expression of pancreatic enzymes.

We provide an integration of the evidence found on legumes’ effects in inflammation, oxidative stress, and metabolism by anatomic site in Figure 3.

## 6. Biotechnology

According to current evidence, bioactive compounds present in legumes represent a promising approach to healthcare. However, certain limitations are inherent due to their nature—they are susceptible to degradation by heat treatment, pH, storage temperature, oxygen, enzymes, and light. Therefore, extraction methods and biotechnological techniques to provide stability are of utmost importance for effectiveness [97]. This section includes the biotechnological aspects of extraction, identification, and quantification of phenolic and anthocyanin compounds, recovered from the methodological research. In that sense, other more detailed approaches focus on this [98] that were not established in the findings of this article.

An efficient extraction method significantly increases the final total concentration of phenolic compounds and anthocyanins. Leaching, also known as conventional extraction, it is the most commonly found in the papers considered in the current review. It is considered a solid–liquid extraction in which organic solvents and acids are used, in addition to specific temperature and shaking. Temperature selection is a crucial aspect to avoid compounds’ degradation, so a range of 40–60 °C is suggested; however, effective extraction has been observed as low as 25 °C [99,100]. Methanol has been used as a solvent in certain studies; however, ethanol is preferred to provide food-grade extraction. Regarding acids, the most widely used are hydrochloric, acetic, citric, and formic acids. The acidification solvent is crucial because it promotes greater stability of anthocyanins and phenolic compounds. Methanol acidification generates significantly higher levels of anthocyanins compared to other solvents, and directly, the anthocyanin fractions seem to be involved in the higher antioxidant capacity evaluated by two assays, the ferric ion reducing antioxidant power (FRAP) and oxygen radical absorbance capacity (ORAC) assays [101,102]. Anthocyanins show higher stability in the pH range 1–3, and this decreases as the pH becomes neutral or alkaline [103,104]. Subsequent to extraction, the liquid extract is concentrated to eliminate the solvent, and this process is carried out in a rotary evaporator. Seventeen reviewed articles implemented an intervention with lyophilized extract. This aspect is noteworthy, considering that lyophilization involves extract dehydration to maximize the stability and shelf life of anthocyanins and phenolic compounds in the extract. Only four articles reported an intervention with the liquid extract; in this case, the anthocyanins and phenolic compounds are highly exposed to environmental factors, such as temperature changes, storage time, and pH variations, which increase their susceptibility to degradation. Nevertheless, it all depends on the characteristics and the research objective [82,100,105].

Food biotechnology provides innovative alternatives to conventional extraction. Pressurized liquid extraction (PLE) and supercritical fluid extraction (SFE) are novel options to conventional leaching extraction—both reduce the solvent required and optimize the processing time. The PLE principle is the utilization of solvents at high temperatures coupled with high pressure, which promotes greater penetration of the solvent into solid foodstuffs, whereas SFE employs carbon dioxide in its supercritical state, giving it the advantage of easily dissolving as a liquid and simultaneously permeating into foodstuffs as a gas. This aspect is responsible for increasing the extraction yield of phenolic compounds and anthocyanins by SFE [100]. Several studies have proven the efficacy of SFE in the extraction of phenolic compounds and anthocyanins, not only in quantity but also in antioxidant capacity and biological effect [100,106]. It has been proven by Fonseca et al. that the method of extraction directly impacts the effect that the beans’ anthocyanins may have. When comparing the pure and crude extracts with SFE versus leaching, pure SFE had a better IC_50_ antioxidant capacity, proven on two radical assays (DPPH and ABTS) [107]. Hsieh-Lo et al. also showed a better extraction efficiency with SFE than leaching [100].

Regarding phenol and anthocyanin profile identification and their quantification, one of the most common techniques is HPLC (high-performance liquid chromatography), from which Fraboni et al. used UHPLC-PDA-ESI/MS in two types of spectrophotometry to quantitatively evaluate anthocyanins’ pigments. A wide range of variation in the distribution of anthocyanins compounds was observed, where cyanidin derivatives were present in all the food samples except for black bean. The method of extraction was taken into consideration, where water and ethanol are suitable for food application at 20:80, respectively, and the acid used to stabilize the anthocyanins was citric acid (1%) to reduce acylated anthocyanins’ breakdown [108]. There are some other biotechnological techniques for the analysis and quantification of phenolic compounds and anthocyanins, but they were not reported in the reviewed papers. In case this caught your attention, we suggest the review in [109].

## 7. Conclusions

Phenolic compounds comprise a diverse family of molecules, including anthocyanins, which encompass over 600 distinct compounds. Due to their chemical structure, their primary effects are linked to anti-inflammatory and antioxidant properties, with many studies also highlighting their antidiabetic and anti-obesity potential. However, despite the promising nature of these terms (antidiabetic and anti-obesity), there is no consensus on their precise definitions, leading to a broad range of experimental approaches in different models, predominantly in vivo and in vitro. The collected evidence demonstrated significant effects on various anatomical sites, including the liver, kidney, brain, adipose tissue, muscle, and gastrointestinal and cardiovascular systems.

Regardless of their beneficial attributes, the same chemical structure that confers these properties also renders these molecules highly unstable. As a result, further research is needed to refine methods for extraction, stabilization, and biotechnological modifications to optimize their health benefits and provide clear dietary recommendations.

The phenolic and anthocyanin content in plants varies significantly, with those exhibiting blue, black, and red pigments showing the highest concentrations. Legumes, in particular, present a promising option for anthocyanin and phenolic compound consumption due to their wide acceptance, sustainable production, and growing evidence supporting their positive health effects.

Although there are numerous reports characterizing these molecules in various food sources, the data remain subject to rapid fluctuations. Many variables, including environmental factors, such as light, drought, salinity, and pH, can influence the biochemical pathways responsible for phenol and anthocyanin synthesis, notably, the shikimic acid and phenylpropanoid pathways. The phenolic content may vary not only between different legume species but also among different harvests of the same legume variety.

Regarding absorption, while the small intestine serves as the primary site, the colon plays a crucial role as well, where the microbiota biotransforms these secondary metabolites into more bioactive forms.

Of the legumes analyzed, soybean yielded the most significant results. However, of the 15 articles reviewed, only 1 involved human subjects, underscoring the need to prioritize translating these findings into clinical trials. Preclinical studies remain essential for delineating molecular pathways, gene expression, dosage, and tissue-specific effects. Finally, the main challenges to be addressed include precise dose calculations, routes of administration, stabilization, and enhancing bioavailability through biotechnological advancements.

This comprehensive review revealed the complexity of legumes’ nutritional and health-promoting properties. While numerous studies emphasize anthocyanins and phenolic compounds as primary contributors to antioxidant and anti-inflammatory capacities, legumes contain a diverse array of bioactive compounds, including peptides, saponins, alkaloids, terpenes, and other essential nutrients, which collectively contribute to their medicinal and nutritional value. Future research should go beyond simplistic attributions and focus on comprehensive profiling of bioactive compounds, investigation of synergistic interactions, and elucidation of the molecular mechanisms underlying the potential health benefits of legumes. This approach will improve our understanding of legumes’ biochemistry and could lead to more targeted nutritional and therapeutic applications.

## Figures and Tables

**Table 1:** Synthesis of evidence: metabolic, anti-inflammatory, and antioxidant effects of beans.

Bean Variety	Study Design	Intervention	Dose/Time	Main Results	Health Effect
Navy and black beans [57]	In vivo, C57BL/6 mice (induction of experimental colitis)	Bean flour, administered orally	20% black or navy bean flour/2 weeks	↓ mRNA expression of *Il-6*, *Ifn-γ*, and *Il-22* in both bean diets, *p* < 0.050 ↑ *Il-10* mRNA expression *p* < 0.050 in both bean groups ↓ mucosal expression gene *Cxcl1* and *Cxcl25*, *p* < 0.010 and *p* < 0.050, respectively ↑ expression of *Il-8*, *p* < 0.050, *Tlr4*, *p* < 0.010, and *Fasl p* < 0.010 ↓ colonic mRNA expression of *Il-17A* comparing BD+DSS vs. BBG diet, *p* < 0.050 ↑*Ccl12* and *Cebpb*, *p* < 0.050 and ↓ *Il-9*, *p* < 0.050, seen only in NB diet vs. BD+DSS ↓ serum concentrations of IL-1β, IFN-γ, TNF-α, and IL-7A in both bean diets, *p* < 0.050	Immune and inflammatory response regulation
Red bean [58]	In vitro, RAW 264.7	Lyophilized extract	50–200 µg/mL	⊥ LPS-stimulated nitrite production with 200 μg/mL, *p* < 0.050 ^a^ ↓ mRNA expression of iNOS and *COX-2* in LPS-induced cells by 26% and 34% with 200 μg/mL, *p* < 0.050 ^b^ ⊥ *Tnf* and *Il-6* mRNA expression by 40% and 39%, with 200 ug/mL, *p* < 0.050 ^b^ ↓ ~44% MDA production when treated with 200 μg/mL, *p* < 0.050 ^b^	Anti-inflammatory and antioxidant protection
Black bean [55]	RCT (Adults age > 18 with MS)	Moderate-fat breakfast with (1) BB meal, (2) FM meal or AM meal administered orally	Postprandial response, no dosage reported	↓ insulin response after consumption of BB, obtaining 240 ± 30.9 pmol/L, *p* < 0.050 ^c^	Insulin sensitivity enhancement
Adzuki bean [56]	In vivo, C57BL/6 Mice	Bean powder (administered orally)	10% and 20% Adzuki bean powder/10 weeks	↓ liver, kidney, abdominal, and epididymal adipose tissue weight in both Adzuki groups vs. HCD, *p* < 0.001 ↓ TG (118.10 ± 3.39 and 110.56 ± 2.79 vs. 126.45 ± 4.08 mg/dL), TC (130.29 ± 6.81 and 120.07 ± 5.93 vs. 202.16 ± 25.62 mg/dL), and LDL (200.00 ± 11.55 and 191.43 ± 13.45 vs. 227.14 ± 11.13 mg/dL) serum levels of both 10AB-HD and 20AB-HD groups vs. HCD, *p* < 0.010 ^a^, whereas HDL (41.17 ± 2.44 and 48.78 ± 5.10 vs. 30.52 ± 7.95 mg/dL) increased, *p* < 0.010 ^a^, respectively ↓ Hepatic triglyceride (76.89 ± 4.28 and 50.99 ± 7.64 vs. 200.55 ± 32.41 mg/dL) and hepatic TC (12.72 ± 0.83 and 11.14 ± 0.57 vs. 14.06 ± 0.81 mg/dL) levels in both Adzuki groups compared to the HCD group *p* < 0.010 ^a^ ↑ SOD (105.22 ± 2.31 and 105.75 ± 2.43 vs. 100.52 ± 2.14 U/mL) activity in both groups; GPx (5.18 ± 0.67 and 5.47 ± 0.92 vs. 3.75 ± 0.04 U/mL) and CAT (1.33 ± 0.41 and 2.34 ± 0.37 vs. 0.80 ± 0.39 u/mL) compared to HCD group, respectively, *p* < 0.010 ^a^ ↓ mRNA expression of *Cpt-1*, *Srebp-1c*, and *Fas* in both Adzuki groups, *p* < 0.010 ↑ mRNA expression of *Ppar-α* only in the 20AB-HD group, *p* < 0.010 ↓ mRNA expression of *Ampkα* and adiponectin, *p* < 0.0001 ↑ *Hmgcr* and *ApoB p* < 0.010 seen in both Adzuki groups ↑ mRNA expression of *Casp3* in the 20AB-HD group, *p* < 0.010 ↓ mRNA expression of *Tnf*, *Nf-κb* in both groups, *p* < 0.001 and *p* < 0.010, respectively	Lipid-lowering and anti-inflammatory effect
Black bean [61]	In vitro, test tubes	Lyophilized extract	1 mg/mL	⊥ DDP-IV enzyme being malvidin with 82.4% and delphinidin 34.4%, being higher than the anthocyanin extract 34.4% *p* < 0.050 ^b^	Antidiabetic potential, and antioxidant capacity
	In vitro, Caco-2 cells			↓ glucose uptake at 60 and 180 min compared to untreated control *p* < 0.050 ⊥ ROS with malvidin 91.2% *p* < 0.050 ^b^	
Black soybeans and Adzuki beans [60]	In vivo, WKY/SHR rats	Pulverized extract (administered orally)	(250 and 500 mg/kg)/8 weeks	↓ AST and ALT serum levels in AE250 (10.7 ± 0.72, 7.36 ± 2.32 mg/dL, respectively) and AE500 (10.0 ± 1.31, 8.31 ± 0.82 mg/dL, respectively) vs. SHR (24.7 ± 1.95, 19.3 ± 2.50 mg/dL, respectively), *p* < 0.050 ^a^ ↓ TG, TC, and LDL plasma levels in AE250 (74.5 ± 4.88, 75.8 ± 0.25, 7.70 ± 0.07 mg/dL, respectively) and AE500 (74.9 ± 3.38, 80.3 ± 2.31, 7.80 ± 0.18 mg/dL, respectively) vs. SHR (135 ± 7.24, 95.6 ± 1.21, 9.53 ± 0.44 mg/dL), respectively, *p* < 0.050 ^a^ ↓ SBP in both Adzuki groups since the 2nd week compared to SHR group, *p* < 0.050 ↓ SBP 17% from AE250 and 19% for AE500 at the end ^b^ ↓ DBP of AE250 11% and for AE500 13% in the final week compared to SHR, *p* < 0.050 ^b^ ↓ ACE serum levels for both Adzuki groups, *p* < 0.050 vs. SHR 50 ↓ angiotensin II serum levels in AE500 vs. SHR, *p* < 0.050 ^d^ ↓ mRNA expression of *renin* AE500 (45%) vs. SHR, *p* < 0.050	Liver and kidney function enhancement and antihypertensive effect
Black bean [70]	In vitro (enzymatic inhibition test tubes)	Liquid purified and raw extracts	20 μL/15 min (tyrosinase inhibition assay) 25 μL/30 min (elastase inhibition assay)	↓ IC_50_ for tyrosinase in purified extracts (SFE: 0.147 ± 0.02 mg/mL and LC: 0.143 ± 0.02 mg/mL) vs. raw extracts (SFE: 9.92 ± 1.73 mg/mL and LC: 2.59 ± 0.22 mg/mL) ↓ IC_50_ for elastase in purified extracts (SFE: 0.023 ± 0.07 mg/mL and LC: 0.005 ± 0.01 mg/mL) vs. raw extracts (SFE: 0.142 ± 0.01 mg/mL and LC: 0.105 mg/mL)	Pigmentation decrease and skin aging inhibition effects
	In silico (molecular docking)	N/A	N/A	The phenolic compounds studied (C3G, M3G, D3G, gallic acid, and genistein) presented theoretical free energy values ranging from −5.3 to −7.8 kcal/mol for tyrosinase and −2.4 kcal to −6.8 kcal/mol for elastase.	
Black bean [62]	In vivo, Wistar diabetic rats	Lyophilized extract (administered orally)	260 mg/kg/day for 5 weeks	↓ FBG and TNF-α serum levels in BB group, *p* < 0.050 ^a^ In adipose tissue, significantly differentially expressed genes were found: 406 mRNAs, 39 lnRNAs, and 3 snRNAs ↑ pathways, such as insulin secretion, observing changes from −1.15- to 13.45-fold * ↓ pathways, such as NIK/NF-κB, observing 1.14- to 2.91-fold changes *	Immune and inflammatory response regulation
	In silico analysis	N/A	N/A	–6.2 kcal/mol potential binding capacity of petunidin 3-glucoside to GATA2 and to POUF2AF1	
Black bean [63]	In vivo, Sprague-Dawley/rats	Lyophilized extract (administered orally)	400 mg per kg weight/4 weeks	↓ Glucose intolerance, FBG, insulin, and HOMA-IR in BS and BSR groups, *p* < 0.050 ^a^ ↓ GSP in BS and BSR, *p* < 0.050 ^a^ ↓ TC, TG, LDL, and NEFA and ↑ HDL in serum, in BSR and BS, except the LDL in the BS, compared with the model group, *p* < 0.050 ^a^ ↓TNF-α and IL-1β serum levels in BS (345.47 ± 25.34, 56.06 ± 3.21 pg/mL) and BSR groups (341.76 ± 17.01, 55.71 ± 3.91 pg/mL) compared to the model group (393.13 ± 30.07, 61.88 ± 3.73 pg/mL), *p* < 0.050 ^a^ ↑ IL-6 and IL-10 serum levels in BS (258.28 ± 8.63, 62.17 ± 1.65 pg/mL) and BSR groups (260.22 ± 10.49, 63.83 ± 0.30 pg/mL) compared with the model group (234.98 ± 9.29, 59.58 ± 0.94 pg/mL), *p* < 0.050 ^a^ ↓ MDA and SOD levels in BS and BSR, *p* < 0.050, and CAT ↓ in the BSR group, *p* < 0.050 ^a^ ↓ AST, ALT, and ALP serum levels in the BS and BSR groups when compared with the model group, *p* < 0.050 ^a^ ↓ mRNA expression in liver tissue of *Pi3k*, *Akt*, *Ampk*, *Cpt1*, *Cyp7a1*, and *Ppar-α*, *p* < 0.050, comparing T2DM rats with normal rats ↑ mRNA expression in *Hmgcr*, *G6pase*, and *Pepck* (*p* < 0.050), comparing T2DM rats with normal rats ↓ protein levels of PI3K, AKT, AMPK, HMGCR, G6pase, and PEPCK (only BSR) comparing BS group vs. model group, *p* < 0.050 ^a^ ↓ *α* diversity in the BS group compared to the NC group, *p* < 0.050 ↑ At phylum level, *Verrucomicrobia* in BS and BSR groups, compared to NC, *p* < 0.050 ^a^ ↑ at genus level of *Akkermansia* spp., *Coprococcus* spp., *Phascolarctobacterium* spp., and *Bacteroides* spp. (except BS) in the BS and BSR groups compared with the model group, *p* < 0.010 ^a^ ↓ at genus level of *Bifidobacterum* spp. in the BS and BSR groups compared with the model group, *p* < 0.010 ^a^	Gluconeogenesis and lipogenesis inhibition
Black bean [64]	Crossover RCT, (Adults, age 21–30, BMI 20–29.9 kg/m^2^)	100% Black bean pastas: (1) Knife, (2) Combo-MP, (3) Cyclone-LP administered orally	Postprandial response. No dosage reported	Appetite suppressing presented *p* < 0.05 for all 3 black bean pastas compared to white bread ↓ 0–60 min glucose iAUC comparing whole black beans to white bread *p* < 0.001 ↓ 0–60 min glucose iAUC comparing whole black bean to all pastas, *p* < 0.010 ↑ 0–180 min glucose iAUC of white bread, *p* < 0.010 and knife, *p* < 0.05, compared to whole black beans Time by treatment for serum insulin F (16.8, 214.4) = 4.3, *p* < 0.001. Even more evident between white bread and whole black beans, *p* < 0.010	Food processing determines glycemic and insulin metabolic response
Flor de junio Dalia [68]	In vitro (Enzymatic inhibition test tubes)	Milled cooked beans (Liquid extract)	50–450 μL	Inhibition of digestive enzymes ⊥ of PNLIP of cooked Dalia bean 4.9–11.6%, 50/100 grouped 12% ^b^ ⊥ of α-amylase of groups 50/50 and 100/50, *p* < 0.050 ^b^ ⊥ of α-glucosidase of 50/50 (72%) and 100/50 (154%) groups ^b^	Hypolipidemic and hypoglycemic effects
	In vivo, Wistar/Rat (Obesity Model)	Milled cooked beans (administered orally)	20% (p/p) of cooked beans (obese control, and four treated groups with cooked common bean)/4 months	↓ AUC *p* < 0.05 in all groups except for 50/100 group ^b.^ ↓ TG lowest in 100/50 (33%) than OC, *p* < 0.0001, in the 3rd month, being lower, *p* < 0.050, than 100/100 and 50/50 groups ^b^ ↓ TC in 50/100 and 100/50 groups, *p* < 0.050 after 3rd month ↓ serum cholesterol 22%, TG 71%, LDL 15%, and VLDL 71%, while ↑ HDL 136% compared to OC ^b^ ↓ serum glucose levels 50/100 group 16% compared to OC; however, it was *p* < 0.050 in all groups at the end ^b^	

RAW 264.7: macrophages; Caco-2 cells: human colorectal adenocarcinoma cells; LPS: lipopolysaccharide; iNOS: inducible nitric oxide synthase; COX-2: cyclooxygenase-2; TNF-α: tumor necrosis factor-α; IL-6: interleukin 6; IFN-γ: interferon-γ; IL-22: interleukin-22; Cxcl1: chemokine (C-X-C motif) ligand 1; Cxcl25: chemokine (C-X-C motif) ligand 25; IL-8 interleukin 8; Tlr4: Toll-like receptor 4; FasL: Fas ligand; IL-17A: interleukin 17A; Ccl12: chemokine (C-C motif) ligand 12; Cebpb: CCAAT/enhancer binding protein β; IL-9: interleukin 9; IL-1β: interleukin 1β; BD: Basal diet, NB: BD supplemented with 20% of navy bean; BBG: BD supplemented with 20% of black bean; DSS: dextran sodium sulfate; HCD: high-cholesterol and high-fat diet; Adzuki groups—10AB-HD: 10% Adzuki bean, 20AB-HD: 20% adzuki bean; LDL: low-density lipoprotein; VLDL: very-low-density lipoprotein; HDL: high-density lipoprotein; CAT: catalase; TG: triglycerides; TC: total cholesterol; CPT-1: carnitine palmitoyltransferase I; SREBP-1c: sterol regulatory element-binding protein 1c; PPAR-α: peroxisome proliferator-activated receptor alpha; AMPK-α: adenosine-monophosphate-activated protein kinase α; HMGCR: 3-hydroxy-3methyl-glutaryl-CoA reductase; ApoB: apolypoprotein B 100; SOD: superoxide dismutase; GPx: glutathione peroxidase; CASP3: caspase 3; NF-κB: nuclear factor κB; WKY: Wistar-Kyoto rats, SHR: spontaneously hypertensive rats; BS: bean husk; BSR: bean husk combined with black rice; GSP: glycated serum protein; MDA: malondialdehyde; NEFA: non-esterified fatty acids; IL-10: interleukin 10; PI3K: phosphoinositide 3-kinase; AKT: AKT serine/threonine kinase; CYP7A1: cholesterol 7-alpha hydroxylase; G6pase: glucose-6-phosphatase; PEPCK: phosphoenolpyruvate carboxykinase; NC: normal control group; DDP-IV: dipeptidyl peptidase 4; AST: aspartate transaminase; ALT: alanine aminotransferase; FBG: fasting blood glucose; SBP: systolic blood pressure; DBP: diastolic blood pressure; ACE: angiotensin-converting enzyme; AE250: 250 mg/kg of Adzuki bean extracts; AE500: 500 mg/kg of Adzuki bean extracts; SFE: supercritical fluid extraction; LC: leaching extraction; C3G: cyanidin-3-glucoside; M3G: malvidin-3-glucoside; D3G: delphinidin-3-glucoside; AUC: area under the curve; Groups: 100/50, 50/100, 100/100, 50/50; OC: obesity control; BB: black bean; FM: fiber matched; AM: antioxidant capacity matched meal; LSM: least square means; iAUC: increased AUC; ug/mL: microgram per milliliter; U/mL: units per milliliter; g: gram; mg/dL: milligrams per deciliter; pg/mL: picograms per milliliter; pmol/L: picomoles per liter; ⊥ inhibit; ↓ decrease/downregulate; ↑ increase/upregulate. ^a^ Values are reported on mean ± SD. ^b^ Values are % of change. ^c^ Values in LSM and standard error. ^d^ Values presented in mean ± SEM. * The range integrates other pathways regulated and associated with other effects that are not relevant to this article.

**Table 2:** Synthesis of evidence: metabolic, anti-inflammatory, and antioxidant effects of soybean.

Soybean Variety	Study Design	Intervention	Dose/Time	Main Results	Health Effect
Black soybean [77]	In vivo, Sprague-Dawley rats	Lyophilized extract administered intragastrically	Control group: 1 mL of water Intervention groups: 6 and 24 mg/kg anthocyanins for 40 days	Compared with control group: ↓ Average daily food intake 16.43 g/day in 24 mg/kg group, *p* < 0.010 ^a^ ↓ Body weight gain 18.3 g and 15.01 g in 24 mg/kg and 6 mg/kg groups ^a^ ↓ NPY, GABAB1R, PKA-a, and p-CREB in the hypothalamus with 24 mg treated group, *p* < 0.050	Anti-obesity capacity: Change in daily food intake and body weight gain
Black soybean [82]	In vitro, HaCaT and HNDFs cell lines with 1.5 mM H_2_O_2_	Liquid extract	10, 50, and 100 μg/mL for 24 h	↑ Viability in both cell lines, *p* < 0.050 ↑ VEGF in the HaCaT, *p* < 0.050 ↓ TSP1 in the HaCaT, *p* < 0.050	Wound healing process enhancement
	In vivo, Sprague-Dawley rats with wounds	Liquid extract applied in the wounds	0.1 mL (5 mg/0.1 mL) in the wound or normal saline for 3 weeks	↓ Wound size, *p* < 0.010 ↑ NF-κB cytosol ↓ NF-κB nucleus ↓ IκBα, *p* < 0.050	
Black soybean [80]	In vitro, HLE-B3 cell line under H_2_O_2_-induced oxidative stress	Lyophilized extract	0, 50, 100, and 200 μg/mL (68.3% cyanidin-3-O-glucoside) for 24 h	↓ Cell death in a dose-dependent manner, *p* < 0.010 ↓ BAD, BAX, p53, and caspase-3 with 200 μg/mL, *p* < 0.050 ↑ BCL2 with 200 μg/mL, *p* < 0.050	Cataract prevention
Black soybean [81]	In vitro, DU-145 cells	Lyophilized extract	0, 30, 60, or 120 μM for 24, 48, or 72 h	Compared with control group: ↑ Apoptosis in a dose-dependent manner at all time points, *p* < 0.050 ↑ BAX and p53, *p* < 0.050 ↓ BCL2, *p* < 0.050 ↓ PSA and AR, *p* < 0.050 ↑ NAD+/NADH ratio in the 120 μM treatment for 24 and 48 h, *p* < 0.050	Prostate cancer inhibition and suppression
	In vivo, BALB/c nude mice	Lyophilized extract administered orally	Control group: 1 mL of distilled water for 14 weeks Intervention group: 8 mg/kg for 14 weeks	Compared with control group: ↓ Tumor growth at 8 and 12 weeks post-inoculation, *p* < 0.010	
Black soybean [79]	In vitro, HGECs treatment with high glucose	Extract powder	1, 10, or 50 µg/mL anthocyanin for 6 h	↑ p-AMPK, PPAR-α, PGC-1α, ERR-1α, PPAR-γ, and ACC, *p* < 0.050 ↑ SOD1 and SOD2, *p* < 0.050 ↑ BCL2/BAX, *p* < 0.050	Diabetic nephropathy amelioration via AMPK activation
	In vivo, C57BLKS/J db/m and db/db mice	Extract powder administered orally	Control group: water Intervention group: 10 mg/kg/day for 12 weeks	Compared with control group: ↓ Serum NEFA to 1.04 ± 0.13 mEq/L, *p* < 0.001 ^a^ ↓ TG to 28.0 ± 20.0 mg/dL, *p* < 0.001 ^a^ ↓ TC to 29.4 ± 2.6 mg/dL, *p* < 0.050 ^a^ ↓ 24 h albuminuria to 62.0 ± 37.0 µg, *p* < 0.001 ^a^ ↓ Mesangial fractional area, *p* < 0.010 ↓ TGF-β1, *p* < 0.010 ↓ Type IV collagen, *p* < 0.010 ↑ Levels of PPAR-α and PPAR-γ, *p* < 0.010 ↑ Phospho-AMPK Thr172/total AMPK ratio, *p* < 0.050 ↑ Phospho-ACC/total ACC ratio, *p* < 0.010 ↓ SREBP-1 level, *p* < 0.010 ↑ BCL2/BAX ratio, *p* < 0.010 ↓ TUNEL-positive cells, *p* < 0.010 ↓ 24 h urinary 8-isoprostane concentrations, *p* < 0.010	
Black soybean [75]	In vitro, 3T3-L1 and C2C12 cell lines	Liquid extract	20 μM and 100 μM for 8 days	↑ Adiponectin production to 240% and 425%, respectively, *p* < 0.010 ^b^ ↓ TNF-α production to 57% and 32%, *p* < 0.010 ^b^ ↑ *Pparγ* expression to 180% and 350%, *p* < 0.010 ^b^ ↑ TG level to 115% and 133%, *p* < 0.010 ^b^ ↑ GPDH activity to 126% and 193%, *p* < 0.010 ^b^ ↑ *Cebpa* expression to 150% and 341%, *p* < 0.010 ^b^ ↑ Tyrosine phosphorylation by 1.3-fold and 1.9-fold ↑ *Slc2a4* expression to 208% and 226%, *p* < 0.010 ^b^ ↑ Glucose uptake 132% and 182%, *p* < 0.010 ^b^ Treatment with 100 μM: ↑ *Ppargc1a* expression to 195%, *p* < 0.010 ^b^ ↑ *Sirt1* expression to 140%, *p* < 0.010 ^b^ ↑ *Ucp3* expression to 237%, *p* < 0.010 ^b^	Adipocyte differentiation and insulin sensitivity modulation
	In vivo, BKS.Cg-Dock7m+/+Leprdb/J male mice (db/db mice) and lean mice	Liquid extract administered orally	Control group: water Intervention group: 30 mg/kg/day for 30 days	Compared with control group: ↓ body weight gain, *p* < 0.050 ↓ Total WAT (epididymis, perinephric, retroperitoneum, and intestinal membrane), *p* < 0.010 ↓ Adipocytes’ size ↓ Food intake, *p* < 0.010	
Black soybean [84]	In vivo, C57BL/6N LPS-injected mice	Extract powder dissolved with DMSO intraperitoneally injected	24 mg/kg/day for 14 days (7 days before and 7 days co-treated with LPS)	↓ ROS and MDA levels, *p* < 0.050 ↓ 8-Oxoguanine level, *p* < 0.050 ↓ p-JNK level, *p* < 0.050 In the cortex: ↓ GFAP and ionized Iba-1, *p* < 0.050 ↓ p-NF-kB 65, *p* < 0.050 ↓ TNF-α, IL-1β, and COX-2, *p* < 0.050 ↓ BAX oligomeric form, *p* < 0.050 ↑ BCL2, *p* < 0.050 ↑ Mitochondrial Cyt.C protein, *p* < 0.050 ↓ Cytosolic Cyt.C protein, *p* < 0.050 ↓ Cleaved caspase-3 expression level, *p* < 0.050 ↓ PARP-1, *p* < 0.050 ↓ Number of FJB-positive neuronal cells, *p* < 0.050 ↑ Survival neuronal cells (Nissl staining), *p* < 0.050	Oxidative stress inhibition and neuroinflammation mediation
Black soybean [74]	RCT, age 19–65 years, overweight or obese	Lyophilized extract administered orally in capsule	2.5 g/d capsule (12.58 mg/g extract) 2.5 g/d) or placebo (starch, 2.5 g/d) for 8 weeks	Compared with placebo group: ↓ LDL 122.50 ± 33.71 to 98.47 ± 23.91 mg/dL, *p* < 0.050 ^a^ ↓ Non-HDL mg/dL 171.63 ± 38.08 to 128.78 ± 32.19 mg/dL, *p* < 0.05 ^a^ ↓ LDL/HDL 2.21 ± 0.64 to 2.02 ± 0.60, *p* < 0.01 ^a^	Plasma lipid profile improvement
Black soybean [78]	In vivo, male ICR mice	Isolation of procyanidins oligomers (PA2, PA3, PA4-2, and PA4-1) from ChoronoCare, Fujicco administered orally	Control group: water alone (5 mL/kg body weight) 10 μg/kg body weight for single administration	Compared with control group: ↑ GLUT4 translocation with PA2, PA3, and PA4-2 by 195%, 213%, and 232%, respectively, *p* < 0.050 ^b^ ↑ p-PI3K, *p*-AKT1 at serine 473 with all procyanidin oligomers, *p* < 0.050 ↑ AKT1 at threonine 308, p-IRS-1 and plasma insulin with PA 4-2, *p* < 0.050 ↑ p-AMPK with all procyanidins oligomers in a polymerization-degree-dependent manner, *p* < 0.050 ↑ Adiponectin level with all procyanidin oligomers, *p* < 0.050 ↓ Glucose level with pre-administration of PA 4–2 at 15, 30, and 60 min after the glucose loading, *p* < 0.050 ↓ Postprandial plasma glucose with all procyanidin oligomers, *p* < 0.050	Postprandial hyperglycemia improvement
Black soybean [76]	In vivo, C57BL/6 mice	Lyophilized extract administered orally	Control group: Fed with an HFD Intervention group: Fed with HFD plus extract at doses of 200 mg/kg for 12 weeks	Compared with HFD group: ↓ Body weight 13.3%^b^ ↓ TG, TC, LDL, and MDA levels, *p* < 0.050 ↑ Hepatic SOD and GPx activities, *p* < 0.050 ↓ Hepatic lipid peroxidation, *p* < 0.050 ↓ Expression levels of *Tnf*, *Il-6*, *Nfkb*, and iNOS genes, *p* < 0.050	Diet-induced obesity amelioration by alleviating oxidative stress and inflammation
Black soybean [72]	In vitro, HepG2 cell line induced by H_2_O_2_ and palmitate	Lyophilized extract	40, 80, and 120 µg/mL for 24 h 119.18 mg cyanidin-3-O-glucoside equivalents (Cy3GE) per g	↑ CAT by 36.17%, 42.87%, and 48.85%, SOD by 46.80%, 58.89%, and 77.13%, GPx by 30.21%, 58.01%, and 95.15%, respectively, *p* < 0.050 ^b^ ↓ MDA contents by 37.13%, 48.53%, and 55.70%, *p* < 0.050 ^b^ ↓ Activity on α-amylase in a dose-dependent manner (0.25, 0.50, 0.75, and 1.25 mg/mL) when compared with that of acarbose, *p* < 0.050 ↓ Lipid accumulation in a dose-dependent manner, *p* < 0.050	Hyperglycemia decrease by regulating glycogen synthesis and glucose metabolism
	In vivo, Kunming mice	Lyophilized extract administered orally	Diabetic control: HFD Intervention group: 100, 200, and 400 mg/kg/day for 28 days	Compared with diabetic control: ↓ Fasting blood glucose 31.44%, 39.05%, and 47.97%, respectively, *p* < 0.010 ^b^ In the OGTT test 100 and 200 mg/kg/day groups ↓ fasting blood glucose at time points 30 min, *p* < 0.010 ↓ Insulin level by 41.57%, 47.89%, and 46.49%, *p* < 0.010 ^b^ ↓ HOMA-IR 27.04 ± 4.30, 21.44 ± 3.23, and 18.79 ± 2.86, *p* < 0.010 ^a^ ↑ ISI −6.05 ± 0.07, −6.41 ± 0.34, −6.17 ± 0.32, and −6.05 ± 0.44, *p* < 0.010 ^a^ ↑ Glycogen contents both in liver and muscle, *p* < 0.010 ↓ Serum NEFA 2.43 ± 0.18, 2.17 ± 0.17, and 2.13 ± 0.16 mM, *p* < 0.050 ^a^ ↓ Serum TG 1.75 ± 0.77, 1.60 ± 0.31, and 1.36 ± 0.38 mM, *p* < 0.050^a^ ↓ TC 6.52 ± 0.66, 6.34 ± 0.73, and 5.12 ± 0.58 mM, *p* < 0.050 ^a^ ↑ HDL level 7.00 ± 0.14, 7.34 ± 0.17, and 5.75 ± 0.07 mM, *p* < 0.050 ^a^ ↓ GSP level 3.08 ± 0.19, 2.97 ± 0.20, and 2.87 ± 0.18 mM, *p* < 0.050 ^a^	
Black soybean [83]	In vivo, Sprague-Dawley rats with drip infusion of bacterial suspension (*Escherichia coli*)	Lyophilized extract administered orogastric	Both groups treated for 4 weeks with injection of *Escherichia coli* suspension Control group: 1 mL PBS twice a day for 4 weeks Anthocyanins group: Solution at 50 mg/kg twice a day for 4 weeks	Compared with control group: ↓ CFU/g of prostate tissue to 4.035 ± 0.293, *p* < 0.050 ^a^ ↓ CFU/g of urine to 2.477 ± 0.20, *p* < 0.050 ^a^ ↓ Inflammatory cell infiltration to 3.0 ± 0.7, *p* < 0.050 ^a^ ↓ Acinar change level to 3.1 ± 0.5, *p* < 0.050 ^a^ ↓ Interstitial fibrosis to 3.0 ± 0.5, *p* < 0.050 ^a^	Anti-inflammatory and antimicrobial effects in chronic bacterial prostatitis
Black soybean [66]	In vitro, 3T3-L1 cell line	Lyophilized extract	100, 200, and 400 μg/mL during the differentiation of WAT and BeAT	↓ TG accumulation in WAT and BLA, *p* < 0.001 ↓ PPAR-γ and C/EBPα in WAT and BLA, *p* < 0.050 ↓ PPAR-γ at 200 and 400 μg/mL in BLA compared to that in WAT, *p* < 0.001 In BLA: ↑ pAMPKα/AMPKα dose-dependently, *p* < 0.001 ↑ ATGL dose-dependently, *p* < 0.001 ↑ mRNA of *Cpt2*, *p* < 0.010 ↑ mRNA of *Cidea*, *p* < 0.050 ↑ mRNA of *Nrf1*, *p* < 0.050 ↓ The NAD/NADH ratio	Energy metabolism improvement in BeAT and WAT and transdifferentiation from WAT into BeAT enhancement
Black soybean [60]	In vivo, Wistar-Kyoto rats and spontaneously hypertensive rats	Liquid extract administered orally	Control group: Saline Intervention group: 250 and 500 mg/kg for 6 weeks	Compared with control group: ↓ Relative liver weight ↓ AST to 14.4 ± 0.69 and 11.2 ± 0.37 IU/L, respectively, *p* < 0.050 ^a^ ↓ ALT to 12.2 ± 1.46 and 7.47 ± 0.60 IU/L, *p* < 0.050 ^a^ ↓ TG to 83.4 ± 1.58 and 97.2 ± 9.27 mg/dL, *p* < 0.050 ^a^ ↓ TC 79.2 ± 0.86 and 78.8 ± 1.95 mg/dL, *p* < 0.050 ^a^ ↓ SBP 11% and 14%, *p* < 0.050 ^b^ ↓ ACE level, *p* < 0.050 ↓ *Ren* mRNA expression 56% in 500 mg/kg, *p* < 0.050 ^b^	Antihypertensive and lipid-lowering effect
Black soybean [67]	In vitro, 3T3-L1 cell line co-cultured with RAW264.7 macrophages	Lyophilized extract	12.5, 25, 50, and 100 μg/mL	↓ ROS 40% and 60% with 50 and 100 μg/mL, *p* < 0.001 ^b^ ↓ Percentage of F4/80-positive cells to 1.75%, 1.51%, and 1.25% by 25, 50, and 100 μg/mL, respectively, *p* < 0.001 ^c^ ↓ Co-culture-induced increase in NO, MCP1, PGE2, TNFα, and IL-6 with all treatments, *p* < 0.001 ↑ Production of adiponectin 47%, 58%, 63%, and 73% with 12.5, 25, 50, and 100 g/mL, respectively, *p* < 0.001 ^b^ ↓ LPS-induced phosphorylation of JNK and ERK, *p* < 0.010 ↓ Co-culture-induced increase of NEFA release in a dose-dependent manner, *p* < 0.001 ↑ 2-NBDG uptake by 35% and 45% with 50 and 100 μg/mL, respectively, *p* < 0.010 ^b^ ↑ PPAR-γ reaching 67% and 91% of the mature adipocyte level with 50 and 100 μg/mL, *p* < 0.010 ^b^	Adipocyte disfunction and IR modulation

NPY: neuropeptide Y; GABAB1R: γ-amino butyric acid receptor; PKA-a: protein kinase A-a; p-CREB: phosphorylated cAMP-response element-binding protein; HaCaT: immortalized epidermal keratinocyte cell line; HNDFs: human neonatal dermal fibroblasts; H_2_O_2_: hydrogen peroxide; VEGF: vascular endothelial growth factor; TSP1: thrombospondin 1; NF-κB: nuclear factor-κB; IκBα: nuclear factor of kappa light polypeptide gene enhancer in B-cells inhibitor alpha; HLE-B3: human lens epithelial cell line; BAD: BCL2-associated agonist of cell death; BAX: BCL2-associated X; BCL2: BCL2 apoptosis regulator; PSA: prostate-specific antigen; AR: androgen receptor; NAD+/NADH: nicotinamide adenine dinucleotide/nicotinamide adenine dinucleotide + hydrogen; HGECs: human gingival epithelial cells; AMPK: adenosine monophosphate-activated protein kinase; PPAR-α: peroxisome proliferator-activated receptor alpha; PGC1-α: peroxisome proliferator-activated receptor γ coactivator 1 α; ERR-1α: estrogen-related receptor alpha; PPAR-γ: peroxisome proliferator-activated receptor gamma; ACC: acetyl-CoA carboxylase; SOD: superoxide dismutase; NEFA: non-esterified fatty acids; TG: triglycerides; TC: total cholesterol; TGF-β1: transforming growth factor beta 1; SREBP-1: sterol regulatory element-binding protein 1; TNF-α: tumor necrosis factor-α; GPDH: glycerol-3-phosphate dehydrogenase; *Cebpα*: CCAAT-enhancer-binding protein alpha; *Slc2a4*: solute carrier family 2 member 4; *Ppargc1a*: PPARG coactivator 1 alpha; *Sirt1*: sirtuin 1; *Ucp3*: mitochondrial uncoupling protein 3; WAT: white adipose tissue; LPS: lipopolysaccharide; ROS: reactive oxygen species; MDA: malondialdehyde; JNK: c-Jun N-terminal kinase; GFAP: glial fibrillary acidic protein; Iba-1: ionized calcium-binding adapter molecule 1; IL-1β: interleukin 1β; COX-2: cyclooxygenase-2; PARP-1: poly (ADP-ribose) polymerase 1; RCT: randomized clinical trial; LDL: low-density lipoprotein; HDL: high-density lipoprotein; PA2: procyanidin B2; PA3: procyanidin C1; PA 4-2: cinnamtannin A2; PA 4-1: structural isomer of tetramer EC-(4β–6)-EC-(4β–8)-EC-(4β–8)-EC; GLUT4: glucose transporter 4; PI3K: phosphoinositide 3-kinase; AKT1: AKT serine/threonine kinase 1; IRS-1: insulin receptor substrate 1; HFD: high-fat diet; GPx: glutathione peroxidase; IL-6: interleukin 6; iNOS: inducible nitric oxide synthase; OGTT: oral glucose tolerance; HOMA-IR: homeostatic model assessment for insulin resistance; ISI: insulin sensitivity index; GSP: glycated serum protein; CFU: colony-forming units; BLA: beige adipose tissue; ATGL: anti-adipose triglyceride lipase; *Cpt2*: carnitine palmitoyltransferase II; *Cidea*: Cell-death-inducing DFFA-like effector a; *Nrf1*: nuclear respiratory factor 1; UCP1: uncoupling protein 1; BeAT: brown adipose tissue; AST: aspartate aminotransferase; ALT: alanine aminotransferase; SBP: systolic blood pressure; ACE: angiotensin-converting enzyme; *Ren*: renin; NO: nitric oxide; MCP1: monocyte chemoattractant protein 1; PGE2: prostaglandin E2; ERK: extracellular signal-regulated kinase; 2-NBDG: 2-[N-(7-nitrobenz-2-oxa-1,3-diazol-4-yl)amino]-2-deoxy-D-glucose; 3T3-L1: mouse adipocytes; RAW264.7 macrophages; ↓ decrease/downregulate; ↑ increase/upregulate. Values are reported as ^a^ mean ± SD, ^b^ % of control, and ^c^ final %.

**Table 3:** Synthesis of evidence: metabolic, anti-inflammatory, and antioxidant effects of lentils.

Lentil Variety	Study Design	Intervention	Dose/Time	Main Results	Health Effect
Brown lentil [93]	RCT (overweight, obesity and T2DM patients)	LS consumed in daily diet	60 g/day 8 weeks	↓ TG serum levels compared to control group (−4.5% vs. 22.01% difference to baseline, respectively) ^a^ ↑ HDL-C serum levels compared to control group (8.7% vs. 1.2% difference to baseline, respectively, *p* < 0.050) ^a^	Plasma ox-LDL regulation and lipid profile improvement
Lentil (Asterix, redcliff, redbow, greenland, impower, imigreen, improve, redberry) [88]	In vitro starch hydrolysis (physiological digestion simulation)	Lyophilized extract	100 mg 0–120 min	RDS was highest in redcliff lentil (79.6 ± 0.1/100 g) ^b^ and lowest was redberry (63.9 ± 1.1/100 g) ^b^ SDS content was highest in redberry (7.7 ± 1.6/100 g) ^b^	RDS increase and hydrolysis delay
	Crossover RCT (Healthy patients, BMI < 30 kg/m^2^)	Cooked whole lentils, consumed in daily diet	25 g available carbohydrate (170.1–200 g according to the variety) 10 separate sessions, 5–10 weeks	↓ Glycemic response. None of the lentil meals resulted in a glucose spike, compared to the control (white bread), *p* < 0.050 ^c^	Postprandial glucose peaks decrease, gastric emptying delay, and postprandial responses to insulin increase
Black lentil [92]	In vitro CCD-33Co, HT-29, and HCT 116	Lyophilized extract	2.5 mg/mL and 1.0 mg/mL	↓ Cell proliferation (HCT 116: 62.2%) ^b^ compared to other plant extracts, *p* < 0.050 ^c^ ↑ Apoptotic cells (HT-29: 42.5% and HCT-116: 38.3%), ^b^ *p* < 0.050	Cell growth factors’ inhibition and apoptosis induction
Brown lentil [94]	In vivo Sprague-Dawley rats	Lyophilized extract administered orally	4 mL extract + 16 mL water/24 h, 71 days	↓ TC (319.2 ± 41.2 mg/100 g to 224.8 ± 12.5 mg/100 g) ^a^ (−29.6%, *p* < 0.050) ↑ BAs excretion in feces (3463.6 mg/kg to 4474.2 mg/kg) ↑ SCFA, acetic acid (1777 ± 416 to 2680 ± 553 mg/kg, *p* < 0.050) ^a^, propionic acid (668 ± 204 to 985 mg/kg, *p* < 0.050) ^a^, butyric acid (202 to 440 mg/kg, *p* < 0.050) ^a^ ↓ *Staphylococcus *spp. ↑ *Bifidobacterium *spp.	Hypocholesterolemic and prebiotic effect
Green lentil Red lentil [90]	Crossover RCT (Healthy patients, BMI 20–30 kg/m^2^)	(1) Long-grain white rice, (2) Large green lentil + rice, (3) Small green lentil + rice, (4) Split red lentil + rice, (5) Instant white potato, (6) Large green lentil + potato, (7) Small green lentil + potato, (8) Split red lentil + potato	50 g available carbohydrate Five 3 h morning study visits, with a 3- to 7-day washout period	↓ 13.5% ^b^ (green), 21.5% ^b^ (small green), and 20.5% ^b^ (split red) in glycemic response, compared to rice, *p* < 0.050 ↓ 33.8% ^b^ (large green), 33.9% ^b^ (small green), and 35.6% ^b^ (split red) in glycemic response, compared to potato, *p* < 0.050	PPGR reduction
Red lentil [48]	In vitro colonic fermentation (fecal batch-culture fermentation)	Liquid extract	1 mg/mL 72 h Three independent fermentation experiments (feces from three healthy volunteers)	↓ *Lactobacillus *spp., *Enterococcus* spp., *Clostridium* spp., *p* < 0.050. ↑ *Bifidobacterium *spp., *p* < 0.050	General antimicrobial effect
Beluga lentil [65]	In vitro * AML-12 *	Lyophilized extract	100 μg/mL	↑ >2-fold, *Gclc*, *Gclm*, *Cat*, and *Gr *expression, *p* < 0.050 ↑ Enzyme activity of Cat and Gr, *p* < 0.050 ↑ Nrf2 protein expression, *p* < 0.050	Hepatoprotective effect mediated by antioxidant activity
	In vivo BALB/c mice	Lyophilized extract administered orally	400 mg/kg 2 weeks	↓ ALT and AST serum levels, compared to CCl4 (4860.90 ± 2041.11 vs. 9472.38 ± 2755.53 U/L) ^a^ (6010.76 ± 1140.44 vs. 5158.45 ± 2333.14 U/L, *p* < 0.050), ^a^ respectively ↓ TG serum levels compared to CCl4. (148.43 ± 46.97 vs. 245.63 ± 81.19, *p* < 0.050) ^a^	
Green lentil [91]	RCT (waist circumferences ≥ 35 inches females and 40 inches males)	Cooked green lentil (5 prepared midday meals/week; isocaloric but varied in dose of cooked whole green lentils)	0 g, 300 g, and 600 g/week 8 weeks	↓ HOMA-IR on average 1.5 units lower than control (t = −3.5, *p* < 0.010) ^c^ An inverse relationship was observed between meal enjoyment and self-reported hunger (β = −0.16) ^d^, desire to eat (β = −0.51) ^d^, and amount that they could eat (β = −0.38) ^d^	Acute postprandial glucose and insulin response improvement

AML-12: mouse hepatocytes cell line; CCD-33Co: human fibroblast cell line; HT-29: human colorectal adenocarcinoma cell line; HCT-116: human colorectal carcinoma cell line; CCl4: hepatotoxicity model; Gclc: glutamate-cysteine ligase catalytic subunit; Gclm: glutamate-cysteine ligase modifier subunit; CAT: catalase; Gr: glutathione reductase; RDS: rapidly digestible starch; SDS: slow digestible starch; TC: total cholesterol; BAs: bile acid; SCFA: short-chain fatty acids; ALT: alanine aminotransferase; AST: aspartate aminotransferase; Nrf2: nuclear factor erythroid 2-related factor 2; LS: lentil sprouts; TG: triglycerides; T2DM: type 2 diabetes mellitus; HDL-C: high-density lipoprotein cholesterol; ox-LDL: oxidized low-density lipoprotein; RCT: randomized clinical trial; BMI: body mass index; PPGR: postprandial glucose response; HOMA-IR: homeostatic model assessment for insulin resistance; mL: milliliter; U/L: units per liter; hrs: hours; min: minutes; mg: pmol/L; g: grams; kg: kilogram; m: meters; ↓ decrease/downregulate; ↑ increase/upregulate. ^a^ Values are % of change. ^b^ Values are reported as mean ± SD. ^c^ Values are Student’s *t*-test mean differences. ^d^ Beta coefficient.

**Table 4:** Synthesis of evidence: metabolic, anti-inflammatory, and antioxidant effects of chickpea and legume mix.

Chickpea or Other Legume Variety	Study Design	Intervention	Dose/Time	Main Results	Health Effect
Mix of legumes (bean, pea, lentil, chickpea) [95]	In vivo, WKY SHR	SHR assigned to one of six groups: control (SHR-Ctrl); bean (SHR-B; white navy, black, pinto and red kidney); pea (SHR-P; green and yellow); lentil (SHR-L; red and green); chickpea (SHR-C); mixed pulse (SHR-M; combination of beans, peas, lentils, and chickpeas)	Pulse powder was added to the diets at 30% *w*/*w* pulses for 4 weeks	↓ TC, LDL, and HDL levels of rats fed the pulse-based diets compared with those of the WKY and SHR controls, *p* < 0.050 ^a^ ↓ media:lumen ratio and media width of the aorta by the lentil-based diet Pulse-based diets were found to be able to attenuate the rise in BP in the SHR model, *p* < 0.050	Circulating cholesterol levels’ reduction and vascular remodeling
Chickpea [93]	RCT (Adult women, aged 18–65 years)	3 groups: (1) white rice (control), (2) black beans + white rice, and (3) chickpeas + white rice	Carbohydrate content equivalent to 50 g per portion. Each meal was consumed one morning, 7 days apart. Blood samples collected at fasting, and 30, 60, 90, and 120 min post-treatment	↓ Concentrations of postprandial glucose at 60 and 90 min for both groups: black beans with rice and chickpeas and rice compared with control meal with statistical significance, *p* < 0.050 ^a^ ↓ Postprandial glucose at 120 min in response to chickpeas and rice compared to the control meal, *p* < 0.050 ^a^ Legumes promote the release of satiety hormones, such as CCK and GLP-1, hormones that may be responsible for the 31% increase in self-reported satiety	Satiety increase and, therefore, glycemic response reduction
Mix of legumes (yellow pea, green lentil, chickpea, and pinto bean) [96]	RCT (normoglycemic, normotensive adults, BMI: 18.5–29.9 kg/m^2^)	6 treatments, from whole yellow pea, split yellow pea, green lentil, chickpea, and pinto bean, and the control snack was 100% corn; 40% of the corn flour was replaced with flour from one of these legumes	Food intake was measured with a meal consumed at 120 min. BG, insulin, and appetite are measured regularly before (pre-meal, 0–120 min) and after (post-meal, 140–200 min) the meal. Participants consumed 1 treatment per week, tested with 5 days between sessions	At 30 and 45 min, BG was lower (*p* < 0.050) after consumption of pinto bean compared with whole yellow pea and green lentil snacks At 60 min, pinto bean consumption led to lower BG compared with whole yellow pea snacks Pinto bean and chickpea snacks led to lower, *p* < 0.050, pre-meal BG iAUC (110.06 ± 15.4 and 102.0 ± 14.3) ^b^, compared with control, whole yellow pea, and green lentil snacks (148.0 ± 18.6 and 128.0 ± 19.0) ^b^ Consumption of the pinto bean snack led to lower pre-meal iAUC insulin compared with corn control, whole yellow pea, and split yellow pea snacks (1698.0 + 207.0 vs. 2256.0 ± 251.0, 2299.0 + 258.0, 2269.0 + 346.0), ^b^ *p* < 0.050	Postprandial blood glucose decrease
Chickpea [94]	RCT (Healthy participants aged 18–45 years)	CCP (not refined). Three bread rolls baked with 0% (control), 30%, or 60% of CCP, replacing wheat flour Each bread roll with 20 g of no-added-sugar strawberry jam	Three different bread rolls. Three separate study visits in which they consumed randomly 1 of 3 types of bread rolls. Four days washout between each visit	↓ Postprandial glucose response. The consumption of bread with 60% ^c^ of CCP reduced postprandial glucose levels compared to bread with 0% ^c^ The mean difference was 0.866 mM at 30 min (95% CI: 0.336, 1.397) and 1.063 mM at 45 min (95% CI: 0.491–1.640) ↓ iAUC for glucose of 40% after consumption of 30% ^c^ CCP by 41.97 mmol/L/min, *p* < 0.050 Consumption of bread with 60% CCP led to significantly higher GLP-1 and PYY responses compared to bread with 30% and 0% ^c^, *p* < 0.050, with a mean difference of 3101 pmol/L/min GLP-1 and PYY of 3576 pmol/min of mean difference ↑ postprandial amino acid concentrations in 0%, 30%, and 60% CCP breads; 0% CCP and 30% CCP, *p* < 0.050, 0% CCP and 60% CCP, *p* < 0.050, 30% CCP and 60% CCP, *p* < 0.050 ↑ amino acid iAUC with 60% CCP bread consumption compared to 0% CCP bread, *p* < 0.050 ↑ production of amino acids from hydrolysis of proteins	Satiety increase. Glycemia decrease in early postprandial state
	INFOGEST 2.0 (in vitro digestion; enzymatic stimulation of gastrointestinal food digestion)	Each bread was exposed for 1 day to an oral, gastric, and intestinal digestion. Five preparation days with simulated oral conditions (salivary a-amylase, pepsin, pancreatic a-amylase, trypsin, chymotrypsin)		↑ amino acids concentrations in a dose-dependent manner ↑ in 30% and 60% CCP bread compared to after the 0% CCP bread	

RCT: randomized clinical trial; CT: clinical trial; CCP: cellular chickpea powder; GLP-1: glucagon-like peptide-1; PYY: peptide YY; CCK: cholecystokinin; BMI: body mass index; BP: blood pressure; BG: blood glucose; iAUC: area under the curve; SHR: spontaneous hypertensive rats; WKY: Wistar–Kyoto rats; SHR-Ctrl: spontaneous hypertensive rats control group; SHR-B: spontaneous hypertensive rats bean group; SHR-b spontaneous hypertensive rats pea group; SHR-l: spontaneous hypertensive rats lentil group; SHR-c: spontaneous hypertensive rats chickpea group; TC: total cholesterol; LDL-C: low-density lipoprotein-cholesterol; HDL-c: high-density lipoprotein-cholesterol; CVD: cardiovascular disease; ↓ decrease/downregulate; ↑ increase/upregulate; g: grams; C: Celsius; kg/m^2^: kilogram/square meter; mmol/L/min: millimoles per liter per minute; mmol/L: millimoles per liter. ^a^ Mean differences. ^b^ Values are reported as mean and SD and ^c^ % of change.

## Data Availability

Not applicable.
